# Depletion of potassium and sodium in mantles of Mars, Moon and Vesta by core formation

**DOI:** 10.1038/s41598-018-25505-6

**Published:** 2018-05-04

**Authors:** E. S. Steenstra, N. Agmon, J. Berndt, S. Klemme, S. Matveev, W. van Westrenen

**Affiliations:** 1 Faculty of Science, VU Amsterdam, Amsterdam, The Netherlands; 2 Institute of Mineralogy, University of Münster, Münster, Germany; 3 Faculty of Geosciences, Utrecht University, Utrecht, The Netherlands

## Abstract

The depletions of potassium (K) and sodium (Na) in samples from planetary interiors have long been considered as primary evidence for their volatile behavior during planetary formation processes. Here, we use high-pressure experiments combined with laser ablation analyses to measure the sulfide-silicate and metal-silicate partitioning of K and Na at high pressure (*P*) – temperature (*T*) and find that their partitioning into metal strongly increases with temperature. Results indicate that the observed Vestan and Martian mantle K and Na depletions can reflect sequestration into their sulfur-rich cores in addition to their volatility during formation of Mars and Vesta. This suggests that alkali depletions are not affected solely by incomplete condensation or partial volatilization during planetary formation and differentiation, but additionally or even primarily reflect the thermal and chemical conditions during core formation. Core sequestration is also significant for the Moon, but lunar mantle depletions of K and Na cannot be reconciled by core formation only. This supports the hypothesis that measured isotopic fractionations of K in lunar samples represent incomplete condensation or extensive volatile loss during the Moon-forming giant impact.

## Introduction

The alkali elements, including potassium (K) and sodium (Na), are moderately volatile elements and their measured depletions in the mantles of Vesta, Mars and the Moon are considered a cornerstone for planet formation models^1–4^. K and Na depletions in meteoritic samples from Mars and asteroid (4) Vesta are one of the major lines of evidence to argue for extensive volatile loss during their formation or subsequent magmatic events^3,5^. Similarly, the depletions of K and Na in the lunar mantle relative to the bulk silicate Earth (BSE) are interpreted as primary evidence for extensive volatile loss during the Moon-forming giant impact event^2–5^. In addition to its relevance to constraining planetary budgets of volatile elements, the abundance and distribution of the heat producing element K in planets have profound implications for the thermal evolution of planetary bodies including the Earth and Mars^6–9^. For example, K abundance and distribution may determine the onset and duration of core dynamos^6,8^ as well as plate tectonic evolution on Earth^7^.

To date, the metallic cores that segregated from the silicate mantle during early planetary evolution processes in Mars, the Moon and Vesta have generally not been considered as important alternative or additional reservoirs for alkalis. It has long been recognized, through studies of iron meteorites and laboratory experiments, that under specific conditions alkali elements may partition into Fe-rich metals^6,10–17^, but the specific effects of temperature and composition that may affect metal-silicate partitioning of K and Na are not well constrained. Previous studies were hampered by a range of analytical challenges including loss of alkalis during polishing with lubricants, relatively high detection limits of electron microprobe analysis (EMPA), “smearing” effects during sample preparation (leading to artificial enrichment of alkalis in metals by smearing alkali-rich silicate materials onto their surface) and simultaneous variation of multiple variables, prohibiting isolation of the individual parameters that affect K metal-silicate partitioning. Only two studies determined the sulfide-silicate partitioning behavior of Na^11,17^. As a result, it is hard to quantify to which extent these elements may have partitioned into the cores of differentiated planets, moons, and asteroids. Here, we present the first sulfide-silicate and metal-silicate partition coefficients for K and Na at high temperature and high pressure using a newly set up high-precision LA-ICP-MS (Laser Ablation Inductively Coupled Plasma Mass Spectrometry) technique, specifically constraining the individual effects of temperature and composition on K and Na partitioning. We use these results to assess whether K and Na may have (partly) partitioned into the Vestan, Marian and/or lunar core.

## Results

### High pressure-temperature experiments

**Figure 1:**
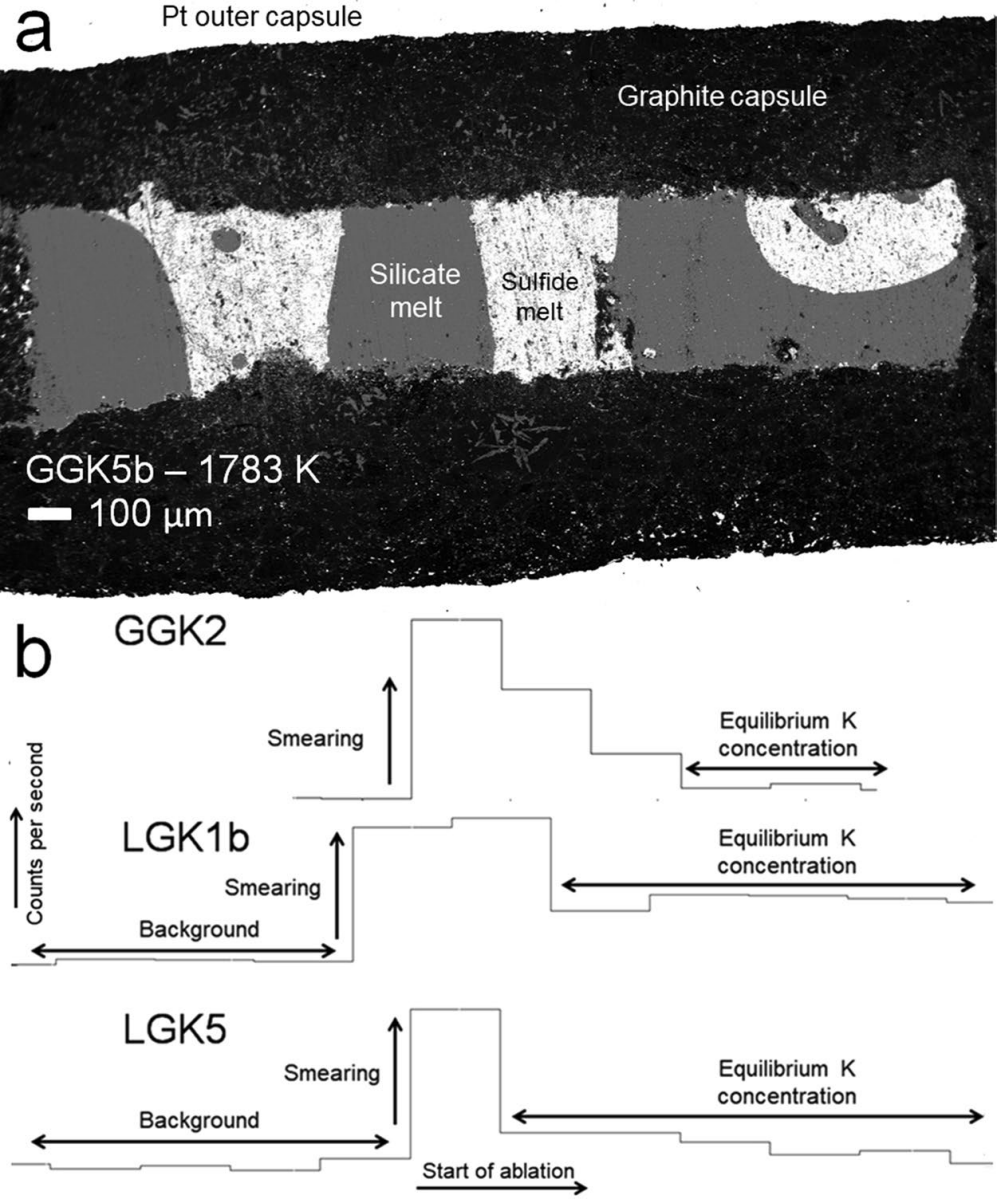
(**a**) Backscattered electron image of typical run product (experiment GGK5b performed at 1783 K, Table 1) (**b**) Example LA-ICP-MS GLITTER® profiles for K in selected sulfides and metals. High signal intensities in the initial ablation reflect the much higher abundances of K on the sulfide or metal surface in some runs due to smearing. The stable plateau after this initial peak reflects the actual concentration of K in the sulfide or metal phase, which is far lower than the initial peak.

Experiments were performed in graphite-lined Pt capsules at a constant pressure of 1 GPa and temperatures of 1683–1883 K to determine metal-silicate and sulfide-silicate partition coefficients for K and Na (Table 1). Starting compositions consisted of synthetic analogues of a primitive basaltic glass and a rhyolitic composition combined with Fe-C, Fe-C-Si or FeS metals. After careful dry polishing^6^, samples were analyzed with electron microprobes (EMPA) at Utrecht University and Münster University and using LA-ICP-MS at Münster University (Supplementary Information). Run products were characterized by well segregated Fe(-S) blobs in a quenched silicate melt (Fig. 1). Experimental run products of the basalt series quenched occasionally to a heterogeneous spinifex-textured glass, whereas the granitic composition always quenched to a glass.

**Table 1:** Experimental run conditions and measured equilibrium constants for K and Na at 1 GPa. All values are based on LA-ICP-MS measurements of both metal and silicate, except for run GGK6 (Supplementary Information). Numbers in brackets are errors in terms of least digits cited and calculated through simple error propagation while assuming 2 standard errors for EPMA and LA-ICP-MS analyses.

Run #	T (K)	Time (min)	Composition	nbo/t^a^	log *K*_K_	log *K*_Na_
GGK1	1683	60	Basalt + Fe	1.95	−2.93(21)^b^	b.d.l.^c^
GGK2	1783	60	Basalt + Fe	2.24	−2.89(34)	b.d.l.
GGK3	1883	60	Basalt + Fe	2.23	−2.40(10)	b.d.l.
GGK4	1683	60	Basalt + FeS	1.25	−3.04(9)	−3.12(9)
GGK5b	1783	30	Basalt + FeS	1.60	−2.61(5)	−2.51(15)
GGK6	1883	60	Basalt + FeS	1.54	−2.25(9)	−2.22(18)
GGK7	1683	60	Basalt + Fe − 17% Si	0.63	−3.95(9)^b^	b.d.l.
GGK8	1783	60	Basalt + Fe − 17% Si	1.05	−4.21(9)^b^	b.d.l.
GGK9	1883	60	Basalt + Fe − 17% Si	1.07	−4.48(29)^b^	b.d.l.
LGK1b	1883	15	Granite + FeS	0.40	−4.25(3)	−2.89(9)^b^
LGK2	1683	30	Granite + FeS	0.07	−4.08(8)	n.d.^d^
LGK3b	1783	15	Granite + FeS	0.12	−4.23(4)	b.d.l.
LGK4b	1683	60	Granite + Fe	0.08	−3.21(6)	b.d.l.
LGK5	1783	60	Granite + Fe	0.35	−3.06(24)	b.d.l.
LGK6	1883	60	Granite + Fe	0.53	−2.84(18)	b.d.l.

^a^nbo/t = [2 × O − 4 × T]/T where [2 × O − 4 T] refers to the number of non-bridging oxygen ions and T represents the number of tetrahedrally coordinated cations^28^ ^b^Close to or below detection limit ^c^Below detection limit ^d^Not determined.

The use of LA-ICP-MS enables significantly more reliable determinations of K and Na abundances in the metal and sulfide phases, as potential “smearing” of K and Na from the coexisting silicate onto the metal can be isolated effectively from the metal signal itself (Fig. 1). For several samples, independent of K content, smearing effects resulted in EPMA significantly overestimating the amount of K, highlighting the necessity of using LA-ICP-MS (Fig. S4, Supplementary Information).

Besides problems with the use of oil-based lubricants during polishing, these “smearing” effects may explain the significant discrepancies between earlier studies. K and Na abundances measured by LA-ICP-MS are not affected by secondary fluorescence and the large beam-size (110 µm) is expected to yield a better average of the metallic composition given the heterogeneous distribution of K and Na in both the metal and silicate melt that is common in these types of experiments (Fig. S1, Supplementary Information).

### Metal-silicate and sulfide-silicate partitioning of K and Na

Partitioning of monovalent K and Na between sulfide/metal and silicate can be described with the following exchange reactions^14^:

1 $$0.5{{\rm{Fe}}}_{({\rm{metal}})}+0.5{{\rm{K}}}_{2}{{\rm{O}}}_{({\rm{silicate}})}\leftrightarrow {{\rm{K}}}_{({\rm{metal}})}+0.5{{\rm{FeO}}}_{({\rm{silicate}})}$$

2 $$0.5{{\rm{Fe}}}_{({\rm{metal}})}+0.5{{\rm{Na}}}_{2}{{\rm{O}}}_{({\rm{silicate}})}\leftrightarrow {{\rm{Na}}}_{({\rm{metal}})}+0.5{{\rm{FeO}}}_{({\rm{silicate}})}$$

where the equilibrium constants (*K*) of the latter reactions can be expressed as:

3 $${\rm{l}}{\rm{o}}{\rm{g}}\,{K}_{{\rm{K}}}=\,{\rm{l}}{\rm{o}}{\rm{g}}\,\frac{{({{\rm{x}}}_{{\rm{F}}{\rm{e}}{\rm{O}}}^{{\rm{s}}{\rm{i}}{\rm{l}}{\rm{i}}{\rm{c}}{\rm{a}}{\rm{t}}{\rm{e}}})}^{0.5}\cdot ({{\rm{x}}}_{{\rm{K}}}^{{\rm{m}}{\rm{e}}{\rm{t}}{\rm{a}}{\rm{l}}\,{\rm{o}}{\rm{r}}\,{\rm{s}}{\rm{u}}{\rm{l}}{\rm{f}}{\rm{i}}{\rm{d}}{\rm{e}}})}{({{\rm{x}}}_{{{\rm{K}}{\rm{O}}}_{0.5,}}^{{\rm{s}}{\rm{i}}{\rm{l}}{\rm{i}}{\rm{c}}{\rm{a}}{\rm{t}}{\rm{e}}})\cdot {({{\rm{x}}}_{{\rm{F}}{\rm{e}}}^{{\rm{m}}{\rm{e}}{\rm{t}}{\rm{a}}{\rm{l}}{\rm{o}}{\rm{r}}{\rm{s}}{\rm{u}}{\rm{l}}{\rm{f}}{\rm{i}}{\rm{d}}{\rm{e}}})}^{0.5}}+\,{\rm{l}}{\rm{o}}{\rm{g}}\,\frac{({\gamma }_{{\rm{K}}}^{{\rm{m}}{\rm{e}}{\rm{t}}{\rm{a}}{\rm{l}}\,{\rm{o}}{\rm{r}}\,{\rm{s}}{\rm{u}}{\rm{l}}{\rm{f}}{\rm{i}}{\rm{d}}{\rm{e}}})}{{({\gamma }_{{\rm{F}}{\rm{e}}}^{{\rm{m}}{\rm{e}}{\rm{t}}{\rm{a}}{\rm{l}}{\rm{o}}{\rm{r}}{\rm{s}}{\rm{u}}{\rm{l}}{\rm{f}}{\rm{i}}{\rm{d}}{\rm{e}}})}^{0.5}}+\,{\rm{l}}{\rm{o}}{\rm{g}}\,\frac{{({\gamma }_{{\rm{F}}{\rm{e}}{\rm{O}}}^{{\rm{s}}{\rm{i}}{\rm{l}}{\rm{i}}{\rm{c}}{\rm{a}}{\rm{t}}{\rm{e}}})}^{0.5}}{({\gamma }_{{{\rm{K}}{\rm{O}}}_{0.5,}}^{{\rm{s}}{\rm{i}}{\rm{l}}{\rm{i}}{\rm{c}}{\rm{a}}{\rm{t}}{\rm{e}}})}$$

4 $$\mathrm{log}\,{K}_{{\rm{Na}}}=\,\mathrm{log}\,\frac{{({{\rm{x}}}_{{\rm{FeO}}}^{{\rm{silicate}}})}^{0.5}\cdot ({{\rm{x}}}_{{\rm{Na}}}^{\mathrm{metal}\,\mathrm{or}\,\mathrm{sulfide}})\,}{({{\rm{x}}}_{{{\rm{NaO}}}_{0.5}}^{{\rm{silicate}}})\cdot {({{\rm{x}}}_{{\rm{Fe}}}^{\mathrm{metal}\mathrm{or}\mathrm{sulfide}})}^{0.5}}+\,\mathrm{log}\,\frac{({\gamma }_{{\rm{Na}}}^{\mathrm{metal}\,\mathrm{or}\,\mathrm{sulfide}})}{{({\gamma }_{{\rm{Fe}}}^{\mathrm{metal}\mathrm{or}\mathrm{sulfide}})}^{0.5}}+\,\mathrm{log}\,\frac{{({\gamma }_{{\rm{FeO}}}^{{\rm{silicate}}})}^{0.5}}{({\gamma }_{{{\rm{NaO}}}_{0.5}}^{{\rm{silicate}}})}$$

where the first term on the right-hand side is the exchange coefficient or ${K}_{{\rm{K}}}^{{\rm{D}}}$, ${K}_{{\rm{Na}}}^{{\rm{D}}}$ and x defined as their molar fraction in the metal or silicate. The second and third terms relate to their activity coefficients (*γ*) in the metal, sulfide or silicate melt^18^. The variability in ${\gamma }_{{\rm{FeO}}}^{{\rm{silicate}}}$ was modeled as a function of silicate melt composition^19^ and ${\gamma }_{{\rm{Fe}}}^{{\rm{metal}}}$ was calculated using the online metal activity calculator^18^ or with a thermodynamic model specifically for Fe-S alloys in the case of S-bearing iron phases^20^ (Supplementary Information). Application of Eqs (3, 4) requires constraints on the activity coefficients of K and Na in the metallic $({\gamma }_{{\rm{Na}},\,{\rm{K}}}^{\mathrm{metal}\,{\rm{or}}\,{\rm{sulfide}}})$ and silicate melts (${\gamma }_{{{\rm{KO}}}_{0.5,}{{\rm{NaO}}}_{0.5}}^{{\rm{silicate}}}$). As there are no predictive models for ${\gamma }_{{{\rm{KO}}}_{0.5,}{{\rm{NaO}}}_{0.5}}^{{\rm{silicate}}}$ as a function of silicate melt composition, we assume ideal behavior^14^. The variability of activity coefficients of K and Na as a function of S and Si in the metal phase were determined using a thermodynamic approach discussed in the next section.

### Effects of metal composition and temperature on K and Na partitioning

To assess the effects of S and Si in the metal or sulfide on sulfide/metal-silicate partitioning, new interaction coefficients were derived using a thermodynamic approach (Supplementary Information). It was found that K and Na behave chalcophile, in agreement with previous studies^6,11,12,16,17^. The addition of Si to the metal has an opposite effect, resulting in a substantial decrease of their siderophile behavior, in agreement with the negative effects of Si on metal-silicate partitioning of virtually all siderophile elements^21,22^. Blanchard *et al*.^14^ recently found no clear effects of S in metal on the metal-silicate partitioning of K at 49–81 GPa and 3600–4100 K. This discrepancy could be related to currently unconstrained effects of temperature and/or pressure on the interaction coefficient between K and S in Fe-S bearing alloys. Such effects could be substantial. For example, Wang *et al*.^23^ found that Cd, In and Zn behave chalcophile at 15 and 20 GPa, whereas Wood *et al*.^24^ reported chalcophobic behavior of these elements at low pressure.

Previous studies found that Fe-S alloys at our experimental conditions should contain up to 1.5 wt.% of O^25–27^, which could affect the partitioning of K and Na. We find that in our experiments, the abundance of O is highly correlated with K, resulting in O concentrations (up to ~5 wt.%) that far exceed the solubility of O predicted for K-free Fe-S alloys at the redox conditions of our experiments (1.44 wt.%)^25,26^ (Fig. S8, Supplementary Information). This suggest that O “follows” K in the sulfide, rather than directly increasing D(K)^17^ values due to its effects on FeO activities in sulfide melts^25–27^, as the O concentrations at very low K contents are within error with that of the predicted O contents for K-free sulfides. Using our derived interaction parameters, we corrected for any variability in the $\mathrm{log}\,{K}_{{\rm{K}},{\rm{Na}}}$ due to different metal compositions (Supplementary Information). Given the geochemical highly similar behavior of K and Na, we assume that the effects of S on Na are the same as that for K^11,17^.

**Figure 2:**
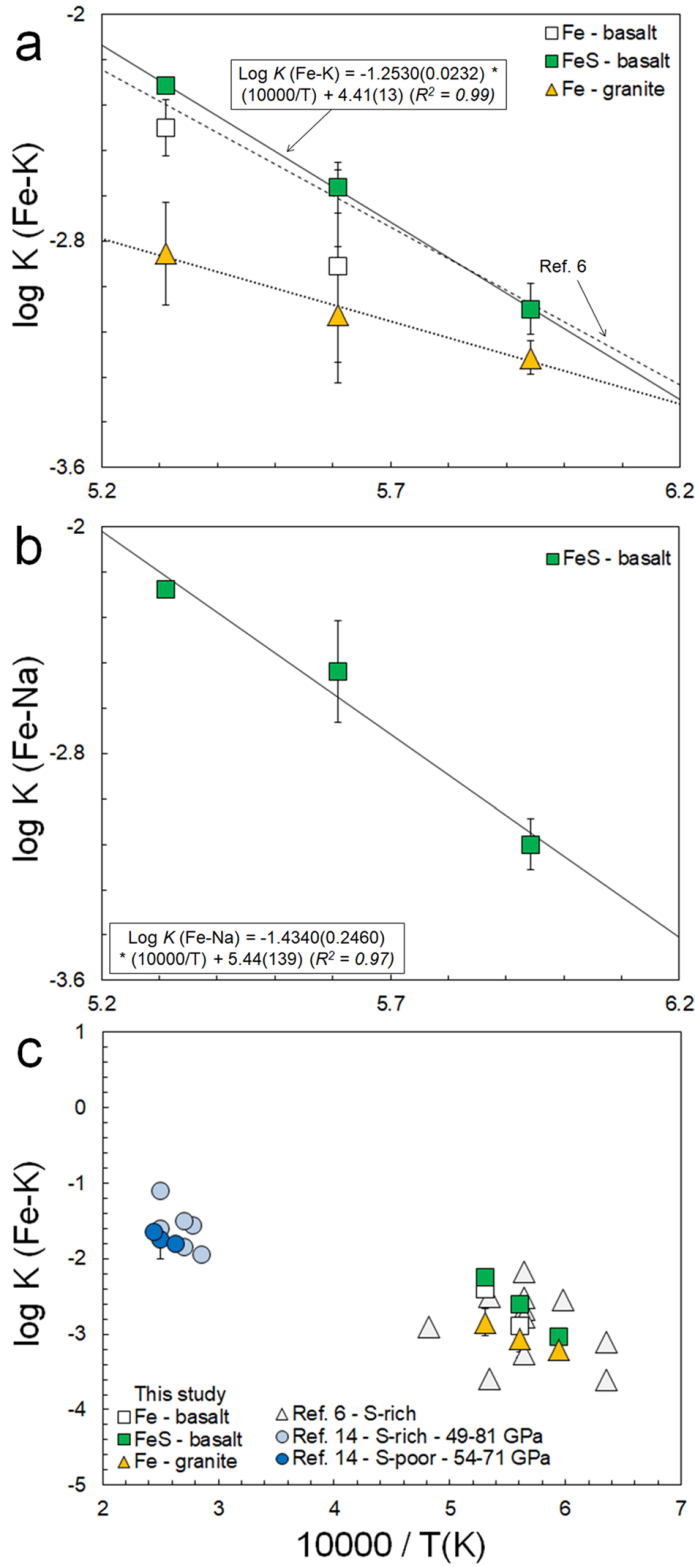
(**a**–**c)** Sulfide-silicate and metal-silicate equilibrium constants for K and Na as a function of temperature (in Kelvin) from this study, Murthy *et al*.^6^ and Blanchard *et al*.^14^. Solid line in (**a**,**b**) are the best-fit dependency for the FeS – basalt data. Errors represent 2 standard errors and were calculated by simple error propagation.

Figure 2 shows our measured sulfide-silicate and metal-silicate $\mathrm{log}\,{K}_{{\rm{K}},{\rm{Na}}}$ values. We find a clear and strong increase of both K and Na partitioning with temperature. The magnitude of the effect found for the experiments with a basaltic silicate melt are in good agreement with two previous studies^6,12^, but much larger than reported in other studies^14,16^. Corgne *et al*.^16^ found no effect of temperature on partitioning of K, which could be related to the use of single capsules in the majority of their experiments, resulting in variable losses of K. The less pronounced effects of temperature within the granitic series are likely related to the high and variable degrees of melt polymerization in that set of experiments (Supplementary Information). Comparing our new sulfide-silicate partitioning data with the recently reported diamond anvil cell data of Blanchard *et al*.^14^ confirms our hypothesis that increasing temperature (and possibly pressure) significantly increases the sulfide/metal-silicate partition coefficient of K. We note that the equilibrium constants from Blanchard *et al*.^14^ derived for <10 wt.% S bearing alloys are likely lower limits relative to our sulfide-silicate partitioning data if S affects K partitioning at these very high pressures.

The dependencies of $\mathrm{log}\,{K}_{{\rm{K}},{\rm{Na}}}$ on temperature are well quantified using the following relationships derived for Fe-S experiments:

5 $$\mathrm{log}\,{K}_{{\rm{K}}}=4.41(13)+\frac{-12530(235)}{T(K)}$$

6 $$\mathrm{log}\,{K}_{{\rm{Na}}}=5.44(139)+\,\frac{-14340(2460)}{T(K)}$$

Pressure may also affect alkali partitioning behavior, but most previous studies found no significant effects of pressure^6,12,15^ (Supplementary Information). Blanchard *et al*.^14^ reported a pressure term for $\mathrm{log}\,{K}_{{\rm{K}}}$ derived over a 70 GPa range. If this term is indeed correct, extrapolation of our 1 GPa experiments to the core-mantle boundary of Mars (~20 GPa), would only result in an increase of ~0.5 in $\mathrm{log}\,{K}_{{\rm{K}}}$. We therefore consider our data to be appropriate for core formation scenarios in bodies ranging from asteroid to at least Mars sized. Alternatively, one can consider our results to be a lower limit.

It has been suggested that polymerization of silicate melts affects the sulfide-silicate and metal-silicate partitioning of K and Na^6,11^. Consideration of $\mathrm{log}\,{K}_{{\rm{K}}}$ versus nbo/t, a single term proxy for the degree of silicate melt polymerization^28^, suggests the iron-loving behavior of K may be affected at the highest degrees of melt polymerization (low nbo/t, see Supplementary Information). We find no consistent variation of log $\mathrm{log}\,{K}_{{\rm{K}}}$ within the range of high nbo/t values relevant for planetary mantles (~2.5–3), consistent with previous studies^6,12,29^. Given the geochemically similar behavior of K and Na, it is likely Na will behave similarly^11^. We therefore apply our results for the basalt-series for modeling core formation in Vesta, Mars and the Moon.

### Assessment of K and Na contents in planetary cores

Many previous studies focused on geochemical and geophysical properties of Mars and Vesta reported high sulfur (S) contents for both of their cores, ranging between 10–25 wt.%^30–35^. High core S contents are inferred from the high abundance of S in their most plausible building blocks and meteoritic samples from these bodies^31,33,36–38^, the existence of core dynamos in their early history^39,40^, depletion patterns of refractory siderophile elements^30,41,42^ as well as geophysical constraints such as their moment of inertia and corresponding core densities^34,43^.

Our new metal-silicate partitioning models for K and Na (Eqs 5, 6) suggest that the measured depletions of K and Na in Martian mantle samples can be completely explained by their partitioning into a 25 wt.% S-bearing Martian core at temperatures of ~2560 ± 60 K and ~2300 ± 200 K, respectively. This is increased to ~2875 ± 75 K and ~2600 ± 250 K for the lower limit of current S contents of the Martian core (10 wt.%). These temperature estimates are in or within <400 K of the range of the temperatures previously proposed for Martian core-mantle differentiation based on non-volatile siderophile element depletions in Martian meteorites^41,42^. Global melting of Mars, possibly extending to the core-mantle boundary, is also supported by the estimated volume of the Martian crust and from the existence of two geochemically distinct source regions required for Martian meteorite genesis^44^. It is also in agreement with the inferred early accretion of Mars from Hf-W-Th isotopic evidence, during which radiogenic heating of ^26^Al decay would already provide sufficient heat to produce a global Martian magma ocean^45,46^ (Supplementary Information).

**Figure 3:**
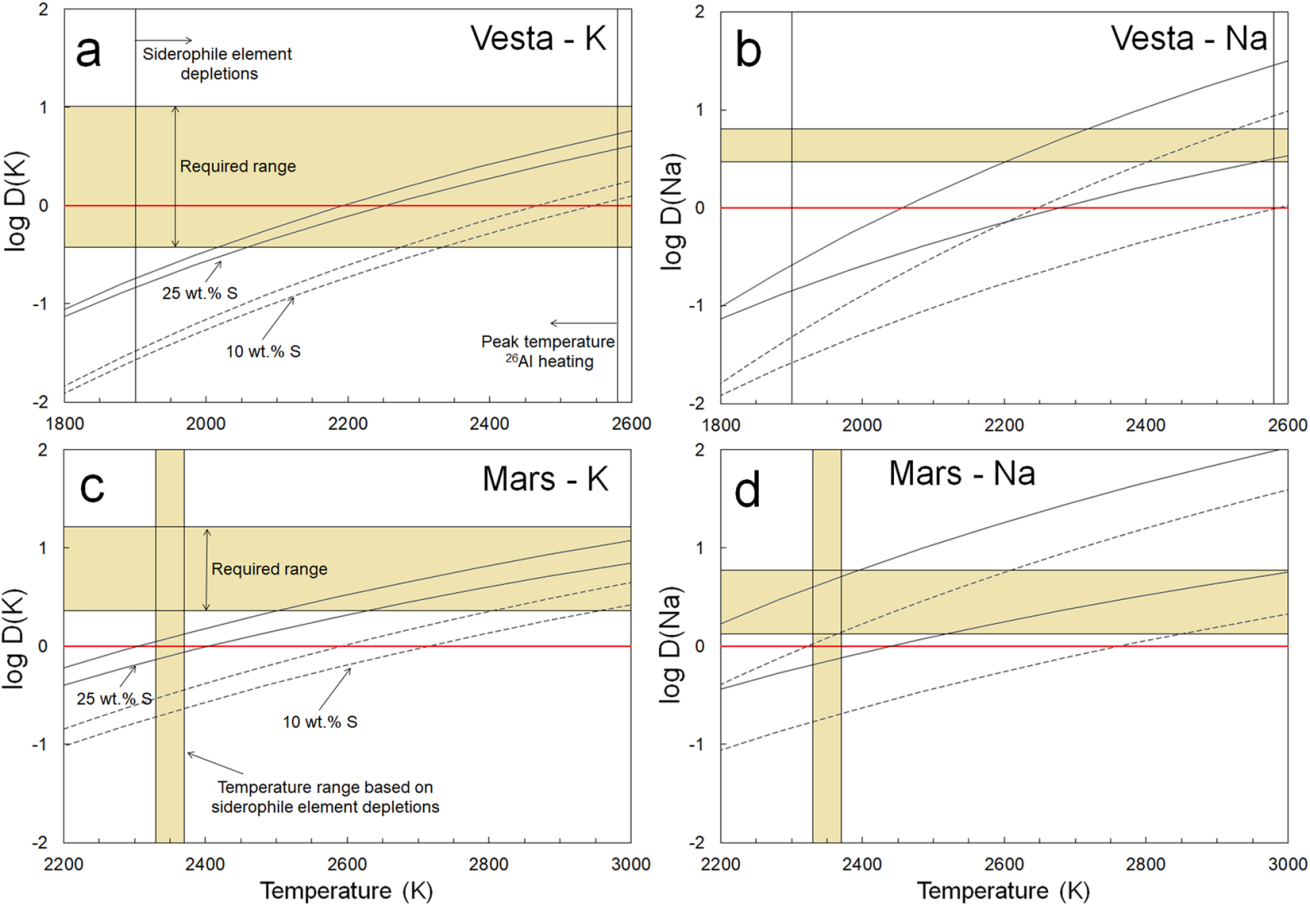
(**a**,**b**) Calculated K and Na partition coefficient ranges for asteroid Vesta, assuming the formation of a 20 to 30 mass % core at −2.2 ± 0.2 log units below the iron-wüstite buffer^30,52,83^, (**c**,**d**) Comparable calculations for Mars, assuming the formation of a 20 to 25 mass% core at −1.25 ± 0.25 log units below the iron-wüstite buffer^34,41–43^, for different core sulfur abundances. Ranges include propagated errors on temperature terms. Plotted for reference are the inferred peak temperature due to radiogenic decay of ^26^Al for Vesta^49^ and core formation temperatures inferred from siderophile element depletions for both bodies^30,41,42,52^. Data sources used to calculate observed alkali element depletions in Mars and Vesta are listed in the Supplementary Materials file. Red line in bold marks the transition from lithophile to siderophile behavior (log *D* = 0).

The Vestan mantle depletions of K and Na can be fully explained by core formation depletion at temperatures exceeding ~2025 ± 25 K for K and ~2370 ± 170 K for Na, assuming a Vestan core with 25 wt.% S (Fig. 3). For a Vestan core with 10 wt.% S, temperatures have to be increased to ~2300 ± 25 K and 2650 ± 250 K to explain all mantle depletion by core segregation, respectively. Such temperatures are fully consistent with the global melting of Vesta implied from numerical thermal evolution models in conjunction with the very early accretion of Vesta in the early solar system when ^26^Al was still abundant^47–49^, homogeneous oxygen isotopic signatures of meteoritic samples from Vesta^50,51^ and siderophile element depletions^30,52^ (Supplementary Information). Such high temperatures would also reproduce an iron isotopic composition of the Vestan mantle that would be consistent with Vesta’s chondritic nature^53–57^.

The temperatures at which the Vestan and Martian K and Na mantle depletions would be fully explained with core formation only are higher than core formation temperatures previously inferred from refractory siderophile element depletions^30,41,42,52,58^. In the Supplementary Information section, we show that refractory siderophile element depletions can also be reconciled with core formation at the higher temperatures required for explaining all alkali depletions, because metal-silicate partition coefficients for refractory siderophile elements are mainly a function of oxygen fugacity, and to a lesser extent core composition in this case^30^. In case of Mars, the higher temperatures would imply whole Mars-melting. This does not agree with thermal constraints on Martian differentiation derived from siderophile element depletions in Martian meteorites that suggest only partial melting of Mars during core formation^41,42,58,59^. It is therefore likely that the higher core formation temperatures derived here indicate that core formation depletion is not the only process depleting K and Na during differentiation of Mars. This is further substantiated by other geochemical lines of evidence discussed later in this section.

Our data show that at temperatures and compositions needed to explain non-volatile element mantle depletions in both Mars and Vesta, alkalis also sequester into their cores in significant proportions. Assuming the minimal partition coefficients of K and Na required to fully explain their Martian mantle depletions, at least ~7250 ppm K and ~1475 ppm Na reside in the Martian core. The same calculation for Vesta yields a minimum of ~260 ppm K and ~3150 ppm Na. Given the geochemically similar behavior of Rb and Cs to K and Na^11^, it is expected that these elements would also partition significantly into metallic cores^11^. Sequestration of alkalis into planetary cores can also explain the observed well-defined correlations between alkalis and incompatible, lithophile trace elements in samples from these bodies that are hard to explain by significant degassing of alkalis (Fig. S10, Supplementary Information). Alkali segregation is also consistent with a lack of post-core formation magmatic devolatilization in bulk silicate Vesta that is suggested by the Zn, Li and Cd stable isotopic compositions of HED’s and the δD ratios values found for most eucrites^60–63^.

The Mn/Na ratios of both the Martian and Vestan mantles are supra-chondritic relative to the Mn//Na ratios of their inferred chondritic bulk compositions^32,64,65^. As Mn behave mores siderophile relative to Na (and K) under the experimental conditions studied here (Tables S3, S4), these ratios require that some Na must have been lost during their early evolution. In addition, Tian *et al*.^66^ recently reported new heavy K isotopic measurements of HED meteorites, which also suggest some amount of K must have been lost before or during Vesta’s differentiation. This agrees with the heavy Rb stable isotopic compositions of HED’s^3^. Alternatively, the data may imply that the Martian and Vestan building blocks were already depleted in Na and K. For Vesta, an initial depletion of Na has been shown to be one of the requirements to produce Juvinas-like eucritic liquids from a chondritic bulk Vesta composition^36^. We note that this does not change the observation that significant quantities of alkali elements can partition in their cores.

In case of the Moon, it is impossible to reconcile Na and K depletions with core formation depletion, unless extremely high core formation temperatures are considered (>3400 K for K and >3600 K for Na, the exact temperature dependent of the assumed core composition and core mass). This is due to the extreme depletions of alkalis in the lunar mantle, relative to bulk silicate Earth (BSE)^67^, and the S-poor nature of the lunar core^68,69^. This observation agrees with the observation of heavier–than-BSE K, Rb, Ga and Zn isotopic compositions of primitive lunar materials, consistent with their evaporative loss from Moon-forming materials^2,3,70,71^. It is also in agreement with the much lower Mn/Mg and similar Mn/Na ratios of the Moon, relative to Mars and Vesta, suggesting Na (and likely other alkalis) were lost in significant quantifies during the Moon-forming event^64,65^. Our results therefore provide additional evidence for evaporative loss of volatiles during lunar formation that support a giant impact origin of the Moon^72–77^.

As illustrated by the lunar example, the point of our work is not to claim that alkali depletion through partial volatilization or incomplete condensation cannot or does not occur. In fact, some loss of alkali elements is required from the K and Rb isotopic signatures of HED’s^66^ and the eucrites Juvinas and Stannern^3^ and δ^37^ Cl isotopic signatures of eucrites^78^ in the case of Vesta, and from Mn/Na ratios in the case of the Moon, Mars and Vesta^64,65^. But our work does indicate that assigning all mantle depletion of alkalis to partial volatilization or incomplete condensation is incorrect under a wide range of planetary formation and core-mantle differentiation conditions. Finally, we note that the heat from radioactive decay of the inferred substantial concentrations of K in the cores of Mars and Vesta may provide a feasible mechanism to generate and sustain the enigmatic early core dynamos in these bodies, suggested by the magnetization of their oldest rocks^79^.

## Methods

### High pressure experiments

Experiments were performed at 1683 K to 1883 K at 1 GPa in a Bristol-type end-loaded piston-cylinder press at the Vrije Universiteit Amsterdam. Metal-silicate and sulfide-silicate partitioning experiments were performed using graphite capsules, which were placed in platinum (Pt) capsules. The Pt capsules were then crimped and welded shut in a triple junction pattern to prevent in- or exfiltration of volatile species. Capsules were placed within a half-inch diameter talc-pyrex cell assembly. Starting compositions were a synthetic analogues of a primitive basalt^80^ and a granitic composition^81^ to study the possible effects of silicate melt composition (Supplementary Table 1). Metal mixtures consisted of pure Fe, Fe_83_Si_17_ or FeS powders. Temperature was monitored using a Type D (97% W/3% Re − 75% W/25% Re) thermocouple and Eurotherm 2404 programmable controller. The center of the samples was located in the hotspot of the assembly, 2 mm away from the thermocouple tip, so that sample temperatures were within 10 °C of the thermocouple reading^24^. Pressure was gradually increased during heating (hot piston-in technique). Experiments were run between 15 and 160 minutes which has previously shown to be sufficient for attainment of equilibrium^16^. Experiments were rapidly quenched by shutting off the power to the furnace. Recovered samples were mounted in petropoxy resin, carefully dry polished to a fine using silicon carbide sandpaper plus graphite powder and subsequently analyzed using EPMA and LA-ICP-MS. Great care was taken to avoid K loss, by preventing any contact with water or oil, and by polishing as close as possible in time before EPMA analyses^6^.

### Analytical techniques

After samples were carbon-coated, major element abundances in the silicate and metal were measured using a JEOL JXA-8800M Electron Microprobe at Utrecht University and a JEOL JXA-8900 Electron Microprobe at the University of Münster (Supplementary Tables 2 and 3). Analysis was done using an accelerating voltage of 15 kV. A 5 µm sized beam was used for homogeneous phases and a 15 µm diameter beam for heterogeneous phases. Metal standards for electron microprobe analyses consisted of tephroite for Mn, chalcopyrite for S, jadeite for Na, KTiPO_5_ for K, MgO for O and pure metal standards for Cr, Fe, Ni. Silicate analyses were calibrated with diopside for Si and Ca, forsterite for Mg, corundum for Al, hematite for Fe, tephroite for Mn, KTiPO_5_ for K, TiO for Ti, jadeite for Na, chalcopyrite for S and pure metal standards for Cr and Ni. Oxygen was measured on the LDE1-multi-layer-crystal at Münster University. Measurements of O were optimized by discrimination of the 2^nd^ order Na K-alpha interference on O by optimizing PHA-settings, by using standards and unknowns with the same coating thickness and through the use of an Evactron plasma cleaner to remove hydrocarbons. Data was processed using the ZAF algorithm^82^.

Laser ablation inductively coupled plasma mass spectrometry (LA-ICP-MS) was used to quantify the abundances of K, Na and other elements in the metallic and silicate melt (Supplementary Tables 2 and 3). K and Na *in-situ* trace element concentrations were measured in high resolution mode ((m/z)/Δ(m/z) = 10,000 at 10% peak valley definition) in order to resolve polyatomic and doubly charged ion interferences (i.e. ^1^H^38^Ar on ^39^K). An 193 nm excimer laser (Analyte G2, Photon Machines) was set to a repetition rate of 10 Hz at a fluence of 3–4 J/cm^2^ for all analyses. Beam size ranged between 50 and 110 µm. Groups of about 20 samples were bracketed with three NIST 610 glass measurements which was used as external reference material for metals and silicates. Internal standard elements for silicates and for metals have been previously determined by EPMA (Supplementary Tables 2 and 3). The signal ablation time was 40 seconds for the peak and 20 seconds for the background. Washout time between individual spots was 15 seconds. Along with the unknown samples a set of well characterized reference materials was analyzed to check for precision and accuracy over the course of this study. Elemental analysis was carried out with an Element 2 mass spectrometer (ThermoFisher) at high resolution mode. Before starting analysis, the system was tuned to get stable signals and high sensitivity, as well as low oxide rates (^232^Th^16^O/^232^Th <0.1%) during ablation. The masses of ^23^Na, ^29^Si, ^39^K, ^43^Ca, ^47^Ti, ^53^Cr, ^55^Mn, ^60^Ni, and ^195^Pt were measured for each spot using the e-scan (i.e. peak jumping) mode. Signals collected from LA-ICP-MS analyses were quantified using the Glitter Version 4.4.2 software. We show in the Supplementary Fig. S3 that there is excellent agreement between measured and recommended and reported K and Na abundances in virtually all reference materials that have been analyzed as unknowns during the course of this study. The abundances in both low K/Na and high K/Na reference materials suggest that the approach used here can be used to adequately quantify K and Na abundances in the experiments reported here.

### Data availability

The datasets generated during and/or analysed during the current study are available from the corresponding author on reasonable request.

## Electronic supplementary material

Supplementary Information File
