# Supplementary material for: Depletion of potassium and sodium in mantles of Mars, Moon and Vesta by core formation
Source: Sci Rep. 2018 May 4;8:7053. doi: 10.1038/s41598-018-25505-6 (PMC5935680; doi:10.1038/s41598-018-25505-6)
Supplement: Supplementary file 1 — Supplementary Information File [file 41598_2018_25505_MOESM1_ESM.docx]

Supplementary information file for: ‘’Depletion of potassium and sodium in mantles of Mars, Moon and Vesta by core formation’’ by E. S. Steenstra, N. Agmon, J. Berndt, S. Klemme, S. Matveev, W. van Westrenen.

**Experimental starting compositions**

Starting compositions were obtained by mixing high-purity powdered oxides and carbonates. Compositions represented a lunar granitic composition and the primitive lunar basaltic Apollo 15 green glass to explore the effects of melt composition on Na and K partitioning^81,82^ (Table S.1). Note that the green glass composition was additionally doped with K_2_O relative to their original compositions, in order for K to remain measurable in FeS-free samples. Both compositions only contained minor amounts of Na_2_O, but sufficient for determining D(Na) for the sulfide-bearing experiments (Table S.2, S.3).

**Polishing techniques**

Because of the high solubility of alkali elements in oil- and water based polishing agents^6^, experiments were dry-polished using graphite powder and different grades of coarse to fine-grained sandpaper (300 to 1400 µm). Samples were polished shortly before each measurement session to prevent possible loss of alkalis to the atmosphere^6^. Note that the latter effects do not affect LA-ICP-MS measurements, as surface contamination is quickly ablated at the onset of ablation.

**Electron microprobe analysis**

Despite careful polishing and calibration of EPMA analyses, some of the silicates show low totals. This is likely due to a combination of some K and/or Na loss upon measurements, presumably due to the high K content of the silicate glasses^29,85^ and/or the rough polished surface which is inevitable for dry-polished samples^6^ (Fig. S.1). To assess the extent of possible alkali loss using EPMA, we compare our results with the K contents derived using LA-ICP-MS. We show in the next section that K abundances in silicate reference materials are reproduced within error. Comparison of K abundances in the silicate melt derived by LA-ICP-MS and EPMA reveals a consistent apparent underestimation of the K contents measured by EPMA. The offset must be a result of alkali loss during EPMA analyses, as the analyses for other elements (Mn, Cr, Ti) agree well (Fig. S.2). This also explains the low totals of many EPMA analyses of the silicates. Assuming LA-ICP-MS values for K instead of EPMA, we find that most EPMA totals are close to 100%. All metal-silicate partition coefficients reported here are therefore based on LA-ICP-MS values for both the silicate and metallic phases, except for run GGK-6 (see section ‘’run GGK6’’).

**Quantification of K and Na abundances using LA-ICP-MS**

K and Na *in-situ* trace element concentrations were measured in high resolution mode ((m/z)/Δ(m/z) = 10,000 at 10 % peak valley definition) in order to resolve polyatomic and doubly charged ion interferences (i.e. ^1^H^38^Ar on ^39^K). An 193nm excimer laser (Analyte G2, Photon Machines) was set to a repetition rate of 10 Hz at a fluence of 3-4 J/cm^2^ for all analyses. Beam size ranged between 50 and 110 µm. Groups of about 20 samples were bracketed with three NIST 610 glass measurements which was used as external reference material for metals and silicates. Internal standard elements for silicates and for metals have been previously determined by electron microprobe (see Tables S2, S3). The signal ablation time was 40 seconds for the peak and 20 seconds for the background. Washout time between individual spots was 15 seconds. Along with the unknown samples a set of well characterized reference materials was analyzed to check for precision and accuracy over the course of this study (Fig. S.3). Elemental analysis has been carried out with an Element 2 mass spectrometer (ThermoFisher) at high resolution mode. Before starting analysis, the system has been tuned to get stable signals and high sensitivity, as well as low oxide rates (^232^Th^16^O/^232^Th < 0.1%) during ablation. The masses of ^23^Na, ^29^Si, ^39^K, ^43^Ca, ^47^Ti, ^53^Cr, ^55^Mn, ^60^Ni, and ^195^Pt were measured for each spot using the e-scan (i.e. peak jumping) mode. Signals collected from LA-ICP-MS analyses were quantified using the Glitter Version 4.4.2 software. We show in Fig. S.3 that there is excellent agreement between measured and recommended and reported K and Na abundances in virtually all reference materials that have been analyzed as unknowns of the course of this study. The abundances in both low K/Na and high K/Na reference materials suggest that the approach used here can be used to adequately quantify K and Na abundances in the experiments reported here.

**Quantification of alkali smearing**

As observed in previous work and despite careful polishing, water-free polishing occasionally results in ‘’smearing’’ of alkalis from the silicate melt onto the metallic surface (Fig. 1 in main text). This potentially results in erroneous alkali metal-silicate partitioning values. This is particularly evident in runs LGK3b, where the K content measured by EPMA is over twice that of several LA-ICP-MS analyses (Table S.3). This must be a result of smearing. We hypothesize that these smearing effects must have contributed significantly to the discrepancies observed between different studies that focused on alkali sulfide-silicate and metal-silicate partitioning. Figure S4 provides a comparison between K abundances in metal derived by LA-ICP-MS and EPMA. Smearing effects are reflected in the significant offsets of K abundances measured with EPMA relative to LA-ICP-MS measurements. The LA-ICP-MS signals reflected the surface contamination (Fig. 1 in main text). High signal intensities upon ablation of the surface reflect the much higher abundances of K on the sulfide or metal surface in some runs. The stable plateau after this initial peak reflects the actual concentration of K in the sulfide or metal phase, which is far lower than the initial peak. The LA-ICP-MS measurements thus provide a valuable independent proxy for assessment of smearing effects.

**The nature of K in sulfide alloys**

Previous studies concluded that it was unclear in which form K occurs in sulfides. Figure S1 shows an elemental abundance map of run GGK4. It is immediately evident that, as previously concluded, K is extremely heterogeneously distributed^6^. The latter study concluded that this must be a result from quenching. The vein-like structures in the sulfides observed here indeed suggest that the heterogeneous K distribution in sulfides is due to quenching. The elemental maps also show that major elements such as Si, Mg and Al are not correlated with the amount of K. It can thus be concluded that the high abundances of K measured in the various sulfides are not the result of incorporation of silicate melt inclusions.

**Run GGK6**

Sample GGK6 was a particularly complex run product where the sulfide strongly interacted with the silicate melt, resulting in occasional presence of silicate melt inclusions (Fig. S.5). Although LA-ICP-MS spots were set in the areas where silicate contamination was not evident on the surface, results suggest soon after initial ablation silicate melt inclusions were incorporated. This resulted in significantly higher abundances of major lithophile elements (e.g., Si, Ca) than would be expected for a sulfide. We therefore use the EPMA value for K and Na of this run. The absence of major lithophile elements (Si, Ca, Al) on the sulfide surface suggested by EPMA data for this run suggests no significant effects of smearing.

**Platinum contamination of sulfide alloys**

Some of the sulfides showed contamination of Pt from the capsule material. This has been observed in studies before^86^ and is one challenging aspect of these experiments. Fortunately, the amount of Pt dissolved in the sulfide liquids is generally limited and is not sufficiently large to significantly affect the alkali metal-silicate partitioning of K and Na.

**Equilibrium**

Previous studies have shown that sulfide-silicate and metal-silicate equilibrium is attained from >15 min at 1673 K for a wide variety of both low and highly charged trace elements^26,87^. As diffusion is greatly increased with temperature, we are confident that our sulfide-silicate and metal-silicate partitioning data reflect equilibrium conditions. For comparison, the run times used here are much longer than the 1-2 minute experiment reported by Corgne et al.^16^ at 250 to 50 K higher temperatures than those reported there. Equilibrium is also suggested from the well-defined trends between data collected at both identical and different run times.

**Equilibrium constants and exchange coefficients for K and Na**

Partitioning of monovalent K and Na between sulfide/metal and silicate can be described with the exchange reactions 1 and 2 in the main text, where the equilibrium constants of the reactions are expressed as main text Eqs. (3, 4)^14^. The variability in $\gamma_{\mathrm{FeO}}^{\mathrm{silicate}}$ was modeled using the expression provided in Wood and Wade (2013)^19^, whereas $\gamma_{\mathrm{Fe}}^{\mathrm{metal}}$ was modeled using the online metal activity calculator^18^ or the model for Fe-S alloys from Lee and Morita^20^. Note that in all of the following calculations we assume that interaction coefficients are pressure-independent within the range considered^14,18,22,24^

Activity coefficients of Fe ($\gamma_{\mathrm{Fe}}^{\mathrm{metal}})$ in Fe-C-Si alloys

The $\gamma_{\mathrm{Fe}}^{\mathrm{metal}}$ values for Fe-C-Si bearing experiments were calculated using the metal activity calculator^18^, accessible via < <http://norris.org.au/expet/metalact/>>. Values for $\gamma_{\mathrm{Fe}}^{\mathrm{metal}}$ are lower than unity ($\gamma_{\mathrm{Fe}}^{\mathrm{metal}}$ ~ 0.72) for all C-saturated experiments, due to the decreasing effects of C on $\gamma_{\mathrm{Fe}}^{\mathrm{metal}}$ (Table S.2). Addition of ~ 15 wt.% Si results in a further decrease in $\gamma_{\mathrm{Fe}}^{\mathrm{metal}}$ to ~0.55.

Activity coefficients of Fe ($\gamma_{\mathrm{Fe}}^{\mathrm{metal}})$ in Fe-S alloy

It has been shown that the metal activity calculator significantly overestimates $\gamma_{\mathrm{Fe}}^{\mathrm{metal}}$ at high S contents (> 10. wt.%)^24^ by up to a factor of 3. To obtain more realistic values, we used a thermodynamic model^20^ for determination of $\gamma_{\mathrm{Fe}}^{\mathrm{metal}}$ in Fe-S alloys. Fig. S.6 shows the variation of $\gamma_{\mathrm{Fe}}^{\mathrm{metal}}$ as a function of the molar fraction of Fe in the Fe-S alloy. The overall $\gamma_{\mathrm{Fe}}^{\mathrm{metal}}$ values calculated for the sulfides range between $\gamma_{\mathrm{Fe}}^{\mathrm{metal}}$ = 1.55 – 1.85, depending of the exact S content and experimental run temperature.

Temperature dependence of interaction parameters

All activity coefficients were based on assuming a reciprocal dependency of interaction coefficient (epsilon values) on temperature. The epsilon values were corrected for different experimental run temperatures according to the following relationship:

$\varepsilon_{i}^{j}\left( T \right)= \frac{T^{0}}{T}\varepsilon_{i}^{j}(T^{0})$ (S.1)

in which *T* is the temperature of interest and *T*^0^ is the temperature (in K) at which the tabulated interaction parameters were derived^18,24^.

Activity of K and Na in Fe-S and Fe-C-Si alloys

Application of main text Eqs. (3, 4) requires constraints on the activity coefficients of K and Na in the metallic $\left( \gamma_{Na, K}^{metal or sulfide} \right)$and silicate melts ($\gamma_{KO_{0.5,}\mathrm{Na}O_{0.5}}^{\mathrm{silicate}}$). There are no predictive models or experimental measurements of $\gamma_{KO_{0.5,}\mathrm{Na}O_{0.5}}^{\mathrm{silicate}}$ to our knowledge, so we are forced to assume unity. Note that we explore the effects of silicate melt composition below, but that these are not significant within the nbo/t range appropriate for planetary mantles (see section ‘’the effects of silicate melt composition).

Experiments were performed using the same metal compositions per subseries, so within these series $\gamma_{Na, K}^{metal or sulfide}$ should be predominantly controlled by addition of either S or Si. Note that there are no quantitative constraints on the effects of C, as all experiments considered here were performed under graphite-saturated conditions. It is expected that C will decrease the activity of K and Na, relative to consideration of a pure Fe alloy^12^. Fortunately, the solubility of C in FeS alloys is extremely low^88^, and will not affect the outcome of this study.

If the compositions of the considered alloys are relatively close the Fe-S or Fe-Si binary and M is present in relatively low levels, the activity coefficient of element M in the metal is closely approximated by^24^:

ln $\gamma_{M}^{\mathrm{metal}}=\ln\gamma_{\mathrm{Fe}}^{\mathrm{metal}}$ + $\ln\gamma_{M}^{0} - \varepsilon_{M}^{S}ln(1-x_{S})$ (S.2)

in the case of Fe-S alloys, and by:

ln $\gamma_{M}^{\mathrm{metal}}=\ln\gamma_{\mathrm{Fe}}^{\mathrm{metal}}$ + $\ln\gamma_{M}^{0}- \varepsilon_{M}^{\mathrm{Si}}ln(1-x_{\mathrm{Si}})$ (S.3)

in Fe-Si allows. In these equations, $\gamma_{M}^{\mathrm{metal}}$ and $\gamma_{\mathrm{Fe}}^{\mathrm{metal}}$ are the activity coefficients of trace element M in the metal phase, $\gamma_{M}^{0}$ is the activity coefficient of M infinitely dilute in liquid Fe, $\varepsilon_{M}^{S}$ and $\varepsilon_{M}^{\mathrm{Si}}$ are the interaction parameter between S, Si and trace element M and X_S_ and X_Si_ refer to the mole fraction of S and Si in the metal alloy, respectively^24^.

Values for $\varepsilon_{K}^{S}$ and $\varepsilon_{K}^{\mathrm{Si}}$can be obtained by consideration of the following equations for S and Si, which are obtained by rearranging the equilibrium constant (main text Eqs. 3,4):

ln $K_{D}=a+0.5 \ln\gamma_{\mathrm{Fe}}^{\mathrm{metal}}- \ln\gamma_{M}^{\mathrm{metal}}$ (S.4)

Substituting for $\gamma_{M}^{\mathrm{metal}}$ using Eq. (S.2) and rearranging then yields^24^:

$\ln K_{D}+0.5 \ln\gamma_{\mathrm{Fe}}^{\mathrm{metal}}=a- \ln\gamma_{M}^{0} + \varepsilon_{M}^{S}ln(1-x_{S})$ (S.5)

The slope of $\ln K_{D}+0.5 \ln\gamma_{\mathrm{Fe}}^{\mathrm{metal}}$ versus $ln(1-x_{S})$ or $ln(1-x_{\mathrm{Si}})$ yields $\varepsilon_{K}^{S}$ and $\varepsilon_{K}^{\mathrm{Si}}$, respectively^24^. Note that in this exercise, $K_{D}$ is defined as the ratio between molar partition coefficient of K or Na ($D_{K}^{*}= \frac{x_{K}}{\mathrm{xK}_{2}O}$) and the molar partition coefficient of Fe ($D_{\mathrm{Fe}}^{*}$)^24^:

$\ln K_{D}$ = $\frac{D_{K}^{*}}{{(D}_{\mathrm{Fe}}^{*})^{0.5}}$ (S.6)

Using the latter approach, we obtain a negative $\varepsilon_{K}^{S}$ value of -7.54(2), in agreement with the previously proposed chalcophile tendencies of K^11^ and a positive $\varepsilon_{K}^{\mathrm{Si}}$ value, suggesting K behaves more lithophile with increasing Si in the metal alloy (Fig. S.7). Unfortunately, the latter approach could not be applied for Na, as Na concentrations are below detection limit in all Fe-rich alloys considered here except for FeS, given its low abundance in the starting materials. We therefore assume that Na behaves in a similar way to K with increasing S in the metal phase, which has been suggested from previous work^11,17^. Note that in the latter calculations we assume a linear dependency of K on the activity coefficient of S and Si in the sulfide/metal. This is justified by the linear dependence of activity coefficients of many other elements in Fe-S bearing alloys^24^. In fact, the assumption of linear dependencies is inherent to the epsilon approach we implement here (and that has been the cornerstone for geochemical core formation models and the steelmaking industry for decades^18,22,24^).

Oxygen may significantly partition into sulfides^25–27^. Whether the K and Na partitioning is increased due to increased partitioning of oxygen into sulfides or that oxygen merely ‘’follows’’ these elements into the metal, can be assessed by calculating the expected amounts of oxygen in the sulfides. Kiseeva and Wood^26,27^ showed that for immiscible sulfides the oxygen content is strongly related to the FeO content of the silicate melt. Our oxygen measurements and those of Murthy et al.^6^ (Fig. S.8) imply significantly higher O contents at similar silicate melt FeO contents within a narrow *T* range. Note that the oxygen in a K-free sulfide is close or within error of that of the predicted level. The discrepancy between predicted and measured O contents increases with increasing K in the sulfide. This shows that K partitioning (and by extension Na ) is not directly dependent on the O contents of the sulfides, but rather that oxygen follows the alkalis into the sulfide melt.

**Effects of silicate melt composition**

Figure S.9 shows the equilibrium constants of K and Na obtained in this study as a function of two common single term parameters for silicate melt polymerization^28,89^ (Table 1 in main text). As observed in Murthy et al.^6^, we find that the equilibrium constant of K decreases with increasing depolymerization of the silicate melt, at low nbo/t (<1). This would be consistent with a decrease in the activity coefficients of K_2_O with increasing SiO_2_ of the silicate melt^29^. This is especially evident in the granitic runs, were log K_K_ consistently decreases with increasing nbo/t. This exercise also demonstrates the great complexity of obtaining meaningful fits when combining multiple datasets into a single set; we find similar temperature and nbo/t dependencies as Murthy et al.^6^, but despite this there is a still some offset between our data and that reported in the latter study. These discrepancies could be related to the high doping levels of K in the latter study, or the use of different analytical techniques. It could also be related to the higher Al_2_O_3_ contents of our experiments, relative to those used by Murthy et al.^6,29^.

**Concentrations of alkalis in planetary bodies**

Basalts from the Moon, Mars and asteroid Vesta show well-established positive correlations of the alkalis (Cs, Rb, K, Na) with incompatible refractory elements^90,91^ (Fig. S. 10). If the alkali abundances were significantly affected by volatility related loss subsequent to formation of these bodies, such correlations would not be expected. Significant post-core formation loss of these elements is therefore unlikely. Hans et al.^92^ also argue that moderately volatile elements were rapidly lost from Vesta within 1 Ma after CAI formation, but suggest they accreted from volatile-poor dust while Rb was not yet condensed (and not incorporated). They found that secondary loss mechanisms, such as devolatilization during accretion, are not required by their Rb-Sr data. We note that ‘’loss’’ of Rb and other alkalis, within 1 Myr, would be compatible with the partitioning of these elements into the cores of Vesta, as suggested from our new experimental partitioning data.

Vesta

For Vesta, we considered a bulk composition ranging between a H chondritic and a H chondritic + 22% CV or 25% CM component, which has been shown to be largely compatible with Vesta’s physical and chemical properties^36^*,* including the Vestan core mass and the depletions of siderophile elements^30^. For the Vestan mantle concentrations of alkali elements, we used the average alkali abundances in non-cumulate eucrite compositions^37^ as well as a recent independent estimate based on remote sensing observations obtained by the DAWN space mission^93,94^*.*

Mars

For Mars, we used the bulk composition reported by Lodders and Fegley^33^ which is based on fitting of the oxygen isotopes of Martian meteorites with mixtures of chondritic meteorites. The advantage of this approach, relative to other approaches is that only one major assumption is required for constraining the bulk composition^32^. Alkali element abundances in the Martian mantle were derived constraints from orbital measurements^32^ and consideration of trace element correlations in the SNC meteorite suite. The correlation between refractory incompatible element U and K is well defined by the following relationship:

K = 10.88(1.70) * U (ppb) + 171(98) (*R^2^* = 0.84) (S.7)

Using the latter equation, we derived a K abundance of 350±158 ppm, assuming an abundance of 16±3 ppb in the Martian mantle^32^. We note that the Martian mantle abundance found for K agrees well with the value proposed by Taylor (2013)^32^, who found 309±36 ppm from remote sensing constraints.

Moon

Alkali element abundances in the bulk Moon (BM) were assumed to be equal to bulk silicate earth (BSE) alkali abundances, given the overwhelming evidence of the isotopic and elemental similarities between the BSE and the BM (Table S.4)^95–97^ Although the Fe difference between BSE and BM was considered to be the only element posing a problem for this hypothesis, a recent study has shown that FeO should also be similar, both in elemental and isotopic view^97^. Therefore, there is no reason to expect that the BSE would be significantly different than the BM. In addition, all current lunar formation models suggest both bodies should have been made from the same reservoir^96^. Lunar mantle abundances of the alkali elements were taken from a wider range of studies and are expected to reflect the uncertainty and overall range in alkali element abundances in the lunar mantle.

**Modeling core formation in asteroid Vesta, Mars and the Moon**

The expected partition coefficients for K and Na relevant for core formation in Vesta, Mars and the Moon were calculated by considering the following equations^98,99^:

log $D_{K}^{*}$ = a + b(1/T) – 0.5 log($x_{\mathrm{FeO}}^{\mathrm{silicate}}/x_{\mathrm{Fe}}^{\mathrm{metal}}$) – log ($\gamma_{K}^{\mathrm{metal}}/(\gamma_{\mathrm{Fe}}^{\mathrm{metal}}$)^0.5^) (S.8)

log $D_{\mathrm{Na}}^{*}$ = a + b(1/T) – 0.5 log($x_{\mathrm{FeO}}^{\mathrm{silicate}}/x_{\mathrm{Fe}}^{\mathrm{metal}}$) – log ($\gamma_{\mathrm{Na}}^{\mathrm{metal}}/(\gamma_{\mathrm{Fe}}^{\mathrm{metal}}$)^0.5^) (S.9)

where a is a constant and b is the temperature coefficient term (see Eqs. 5, 6 in main text). The $x_{\mathrm{FeO}}^{\mathrm{silicate}}$ values were calculated assuming a redox state of ΔIW = -1 to -1.5 for Mars^32,42^, ΔIW = -2.2±0.2 for Vesta^30,52,84^ and ΔIW = -2 for the Moon^100^. The $x_{\mathrm{Fe}}^{\mathrm{metal}}$, $\gamma_{M}^{\mathrm{metal}}\mathrm{and}\gamma_{\mathrm{Fe}}^{\mathrm{metal}}$ terms were recalculated for each modeled temperature for each modeled core composition. Term $\gamma_{\mathrm{Fe}}^{\mathrm{metal}}$ was calculated for Fe-S bearing alloys using the thermodynamic model of Lee and Morita^20^. Parameters $\gamma_{\mathrm{Na}}^{\mathrm{metal}}$ and $\gamma_{K}^{\mathrm{metal}}$ were calculated using our newly derived interaction coefficient for each modeled composition and temperature (Fig. S.7). Due to lack of data for Na in S-poor alloys, we assume $\gamma_{K}^{\mathrm{metal}}$ in Fe-S alloys to be representative for $\gamma_{\mathrm{Na}}^{\mathrm{metal}}$, which is reasonable given the highly similar geochemical behavior of K and Na in these systems observed in this study and previous work^11^. Subtle differences in these interaction coefficients will not change the major outcome of this study, as we show our model is valid across a wide temperature – core composition range. Temperature variability in activity coefficients was modeled using the reciprocal relationship provided in Eq. S.1^18,22,24,99^. The possible effects of oxygen on Fe, K and Na was not taken into account^14^, as we previously showed that O ‘’follows’’ K and does not affect its partitioning. Finally, we assumed the oxide activity coefficient term log (($\gamma_{\mathrm{FeO}}^{\mathrm{silicate}})^{0.5})/(\gamma_{\mathrm{Na}O_{0.5}, KO_{0.5}}^{\mathrm{silicate}}$) to be constant as done in previous work^14^, which is reasonable given the ideal behavior of alkali oxides for compositions close to the basalt used in this study and/or planetary mantles^29^ and the near-ideal $\gamma_{\mathrm{FeO}}^{\mathrm{silicate}}$ values appropiate for planetary mantles^19^.

The required metal-silicate partition coefficients (Table S.5) were then calculated assuming the following mass balance approach^100^:

$D_{\frac{c(i)}{m(i)}}= \frac{C_{BP(i)}-{xC}_{BM(i)}}{C_{\mathrm{BM}}(i)(1-x)}$ (S.10)

where C_BP(i)_ is the concentration by weight of element *i* in the bulk planet, C_BM(i)_ is the concentration by weight of element *i* in the bulk mantle and *x* is the mass fraction of the planetary mantle. In our calculations we assume a core mass of 20-30% for Vesta^30,52,84^, a 20-25% core mass for Mars^34,41–43^ and a 1.5-2.5 % core mass for the Moon^100,101^.

To calculate the resulting depletions of K and Na in the planetary mantles as a function of core sulfur content and temperature (Fig. 3 in main text), we assume full equilibrium between their core and mantle, i.e. in a global magma ocean setting (see next section). Under these assumptions, the resulting core and mantle abundances for K and Na are calculated using the following mass balance equations:

$C_{\mathrm{mantle}}^{i}= C_{bulk planet}^{i}/ [x_{mantle}+\left( 1-x_{mantle} \right)*D\left( i \right)]$ (S.11)

$C_{\mathrm{core}}^{i}= C_{bulk planet}^{i}/ [x_{core}+(1-x_{core})/D(i)]$ (S.12)

where $C_{\mathrm{mantle}}^{i}$, $C_{\mathrm{core}}^{i}$ and $C_{bulk planet}^{i}$ are the concentrations of element *i* in the mantle, core or bulk planet, $x_{core}$ and $x_{mantle}$ represent the core and mantle mass fractions and D(i) represents the metal-silicate partition coefficient for element *i*.

**Evidence for large-scale to complete melting of asteroid Vesta and Mars**

Evidence for very early metal-silicate differentiation of asteroid Vesta is inferred from the presence of excesses of ^26^Mg from the decay of extinct ^26^Al (e.g., in eucrites Piplia Kalan^102^ and Asuka 881394^103^). Current estimates of core formation ages (after CAI formation) in asteroid Vesta range between 4±2.2 Ma^104^ for the ^182^Hf-^182^W system, ${3.7}_{-1.7}^{+2.5}$ Ma for the ^60^Fe-^60^Ni system^46^, whereas Touboul et al.^105^ used the ^182^Hf-^182^W system to propose Vestan core formation already occurred within 1 Ma after CAI formation. The latter age would be consistent with the ^26^Al-^26^Mg differentiation age for Vesta’s upper crust of ${2.66}_{-0.58}^{+1.39}$ reported by Hublet et al.^106^ for basaltic eucrites and the early accretion of Vesta at approximately 0.5 Ma after CAI formation inferred from Sr isotope systematics^92^.

The available heat of radiogenic decay of ^26^Al during Vestan differentiation is highly dependent of the timing and rate of accretion, as well as the initial abundance of ^26^Al present at the time of planetesimal accretion^49^. Considering only ^26^Al heating, numerical models suggest these temperatures could be as high as 2600 to 4000 K^48,49,80^. As a conservative estimate and to reduce extrapolation of our results, we therefore consider temperatures <2600 K. However, the large temperature range illustrate the overall potential of alkali storage in the Vesta core.

Very early accretion of Mars is also required from ^182^Hf-^182^W and ^60^Fe-^60^Ni chronometry on Martian meteorites^45,46^. The latter isotopic compositions suggest Mars accreted approximately 50% of its mass within 2 Ma after CAI formation. In this time, ^26^Al was still extant, which would resulted in global melting^45^. These results also agree with geochemical core formation models for Mars based on siderophile element depletions in Martian meteorites^41,42^.

**Effect of super-liquidus temperatures on siderophile element depletion models for Vesta**

To test whether the high temperatures that would be required to fully explain the K and Na depletions in the Vestan mantle by core formation only are also consistent with previous Vestan core formation models, we ran geochemical core formation models similar to those reported in Steenstra et al.^30^ for refractory siderophile elements. We ran these simulations for different temperatures between 1900 and 2500 K, a pressure of 0.1 GPa (corresponding to the Vestan core-mantle boundary) at -2.2±0.2 log units below the iron-wüstite buffer. As in Steenstra et al.^30^, we considered a H + 22% CV chondritic bulk Vesta composition and considered a 15% wt.% S bearing core. We find (Figure S.11) that increasing the core-mantle differentiation temperature from 1900 K^30^ to 2500 K increases the number of solutions for Mo, Cu and Ga and decreases the number of solutions for P, Ge and Ni. For Co and W, the number of solutions do not significantly change across the whole temperature range considered. Overall, the depletions of all eight siderophile elements are consistent with core formation at temperatures up to 2500 K in Vesta^30^.

**Supplementary references**

85. Siivola, J. On the evaporation of some alkali melts during the electron microprobe analyses. *Bull. Geol. Soc. Finl.* 85–91 (1969).

86. Wykes, J. L., O’Neill, H. S. C. & Mavrogenes, J. A. The effect of feo on the sulfur content at sulfide saturation (SCSS) and the selenium content at selenide saturation of silicate melts. *J. Petrol.* **56,** 1407–1424 (2014).

87. Steenstra, E. S. *et al.* The effect of melt composition on metal-silicate partitioning of siderophile elements and constraints on core formation in the angrite parent body. *Geochim. Cosmochim. Acta* **212,** 62–83 (2017).

88. Tsymbulov, L. B. & Tsemekhman, L. S. Solubility of Carbon in Sulfide Melts of the System Fe-Ni-S. *Russ. J. Appl. Chem.* **74,** 925–929 (2001).

89. Prowatke, S. & Klemme, S. Effect of melt composition on the partitioning of trace elements between titanite and silicate melt. *Geochim. Cosmochim. Acta* **69,** 695–709 (2005).

90. Kreutzberger, M. E., Drake, M. J. & Jones, J. H. Origin of the Earth’s moon: Constraints from alkali volatile trace elements. *Geochim. Cosmochim. Acta* **50,** 91–98 (1986).

91. Birck, J. L. & Allègre, C. J. 87Rb/87Sr study of diogenites. *Earth Planet. Sci. Lett.* **55,** 116–122 (1981).

92. Hans, U., Kleine, T. & Bourdon, B. Rb-Sr chronology of volatile depletion in differentiated protoplanets: BABI, ADOR and ALL revisited. *Earth Planet. Sci. Lett.* **374,** 204–214 (2013).

93. Prettyman, T. H. *et al.* Concentrations of potassium and thorium within Vesta’s regolith. *Icarus* **259,** 39–52 (2015).

94. Prettyman, T. H. *et al.* Elemental Mapping by Dawn Reveals Exogenic H in Vesta’s Regolith. *Science* **338,** 242–246 (2012).

95. Kruijer, T. S., Kleine, T., Fischer-Gödde, M. & Sprung, P. Lunar tungsten isotopic evidence for the late veneer. *Nature* **520,** 534–537 (2015).

96. Zhang, J., Dauphas, N., Davis, A. M., Leya, I. & Fedkin, A. The proto-Earth as a significant source of lunar material. *Nat. Geosci.* **5,** 251–255 (2012).

97. Sossi, P. A. & Moynier, F. Chemical and isotopic kinship of iron in the Earth and Moon deduced from the lunar Mg-Suite. *Earth Planet. Sci. Lett.* **471,** 125–135 (2017).

98. Corgne, A., Keshav, S., Wood, B. J., McDonough, W. F. & Fei, Y. Metal-silicate partitioning and constraints on core composition and oxygen fugacity during Earth accretion. *Geochim. Cosmochim. Acta* **72,** 574–589 (2008).

99. Siebert, J., Corgne, A. & Ryerson, F. J. Systematics of metal-silicate partitioning for many siderophile elements applied to Earth’s core formation. *Geochim. Cosmochim. Acta* **75,** 1451–1489 (2011).

100. Steenstra, E. S. *et al.* The lunar core can be a major reservoir for volatile elements S, Se, Te and Sb. *Sci. Rep.* **7,** 14552 (2017).

101. Weber, R. C., Lin, P.-Y., Garnero, E. J., Williams, Q. & Lognonne, P. Seismic Detection of the Lunar Core. *Science* **331,** 309–312 (2011).

102. Srinivasan, G. 26Al in Eucrite Piplia Kalan: Plausible Heat Source and Formation Chronology. *Science* **284,** 1348–1350 (1999).

103. Nyquist, L. E., Reese, Y., Wiesmann, H., Shih, C. Y. & Takeda, H. Fossil26Al and53Mn in the Asuka 881394 eucrite: Evidence of the earliest crust on asteroid 4 Vesta. *Earth Planet. Sci. Lett.* **214,** 11–25 (2003).

104. Kleine, T., Mezger, K., Münker, C., Palme, H. & Bischoff, A. 182Hf-182W isotope systematics of chondrites, eucrites, and martian meteorites: Chronology of core formation and early mantle differentiation in Vesta and Mars. *Geochim. Cosmochim. Acta* **68,** 2935–2946 (2004).

105. Touboul, M., Sprung, P., Aciego, S. M., Bourdon, B. & Kleine, T. Hf-W chronology of the eucrite parent body. *Geochim. Cosmochim. Acta* **156,** 106–121 (2015).

106. Hublet, G., Debaille, V., Wimpenny, J. & Yin, Q. Z. Differentiation and magmatic activity in Vesta evidenced by 26Al-26Mg dating in eucrites and diogenites. *Geochim. Cosmochim. Acta* **218,** 73–97 (2017).

107. Meyer, C. Lunar Sample Compendium Introduction. *Lunar Sample Compendium* (2009). doi:10.1126/science.caredit.a0800022

108. Jochum, K. P. & Palme, H. Alkali elements in eucrites and SNC-meteorites: No evidence for volatility related losses during magma eruption or thermal metamorphism. *Meteoritics* **25,** 373 (1990).

109. Takahashi, K. & Masudat, A. Young ages of two diogenites and their genetic implications. *Nature* **343,** 540–542 (1990).

110. Mittlefehldt, D. W. The genesis of diogenites and HED parent body petrogenesis. *Geochim. Cosmochim. Acta* **58,** 1537–1552 (1994).

111. Mittlefehldt, D. W., Beck, A. W., Lee, C. T. A., McSween, H. Y. & Buchanan, P. C. Compositional constraints on the genesis of diogenites. *Meteorit. Planet. Sci.* **47,** 72–98 (2012).

112. Steenstra, E. S., Rai, N., Knibbe, J. S., Lin, Y. H. & van Westrenen, W. New geochemical models of core formation in the Moon from metal-silicate partitioning of 15 siderophile elements. *Earth Planet. Sci. Lett.* **441,** 1–9 (2016).

113. Palme, H. & O’Neill, H. Cosmochemical Estimates of Mantle Composition. in *Treatise on Geochemistry: Second Edition* **3,** 1–39 (2013).

114. Taylor, S. R. Refractory and moderately volatile element abundances in the Earth, Moon and meteorites. in *11th Lunar and Planetary Science Conference* 333–348 (1980).

115. Arevalo, R., McDonough, W. F. & Luong, M. The K/U ratio of the silicate Earth: Insights into mantle composition, structure and thermal evolution. *Earth Planet. Sci. Lett.* **278,** 361–369 (2009).

116. Taylor, G. J. & Wieczorek, M. A. Lunar bulk chemical composition: a post-Gravity Recovery and Interior Laboratory reassessment. *Philos. Trans. R. Soc. A Math. Phys. Eng. Sci.* **372,** 20130242–20130242 (2014).

117. Szurgot, M. CORE MASS FRACTION AND MEAN ATOMIC WEIGHT OF TERRESTRIAL PLANETS, MOON, AND PROTOPLANET VESTA. in *Comparative Tectonics and Geodynamics* 5001 (2015).

**Figure S.1.** Elemental maps for K-rich run products showing the heterogeneous distribution of K in sulfides in run GGK4.


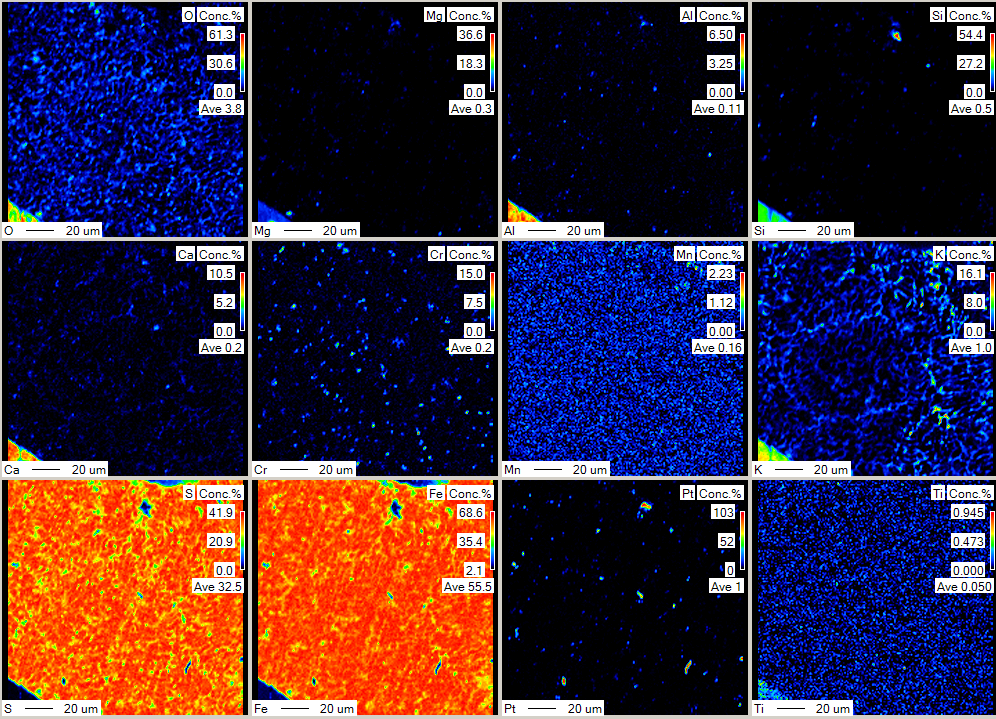


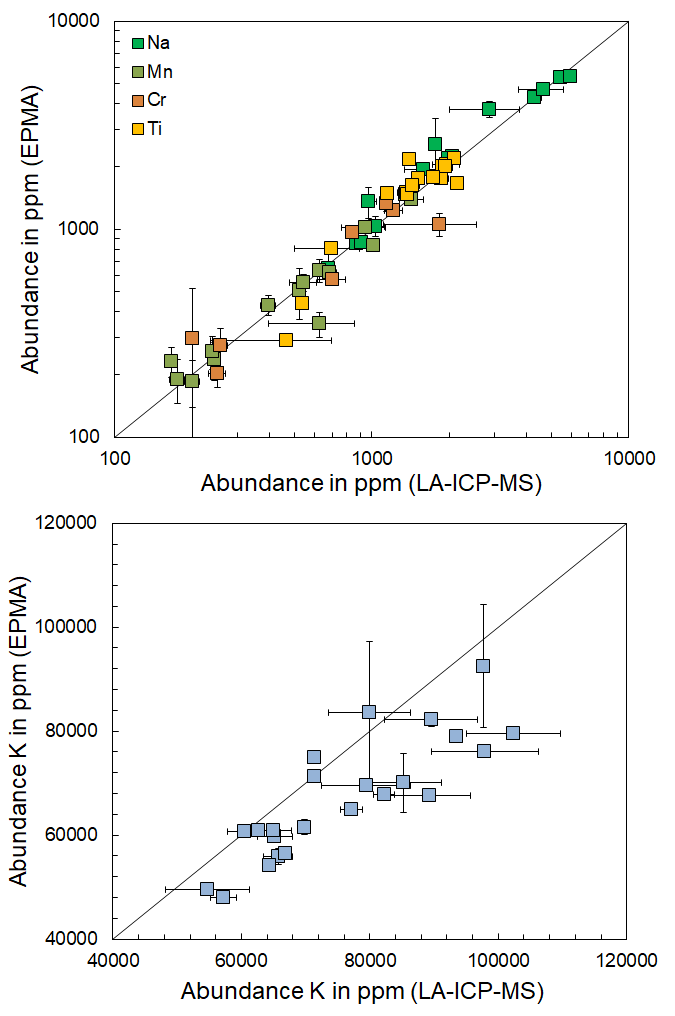


**Figure S.2**. Comparison of Na, Mn, Cr, Ti, K abundances derived with EPMA and LA-ICP-MS. The consistent underestimation of K abundances from EPMA (by up to several wt%) relative to LA-ICP-MS values, in conjunction with the low EPMA totals for these glasses, suggest K volatilization upon EPMA measurements^29,85^.


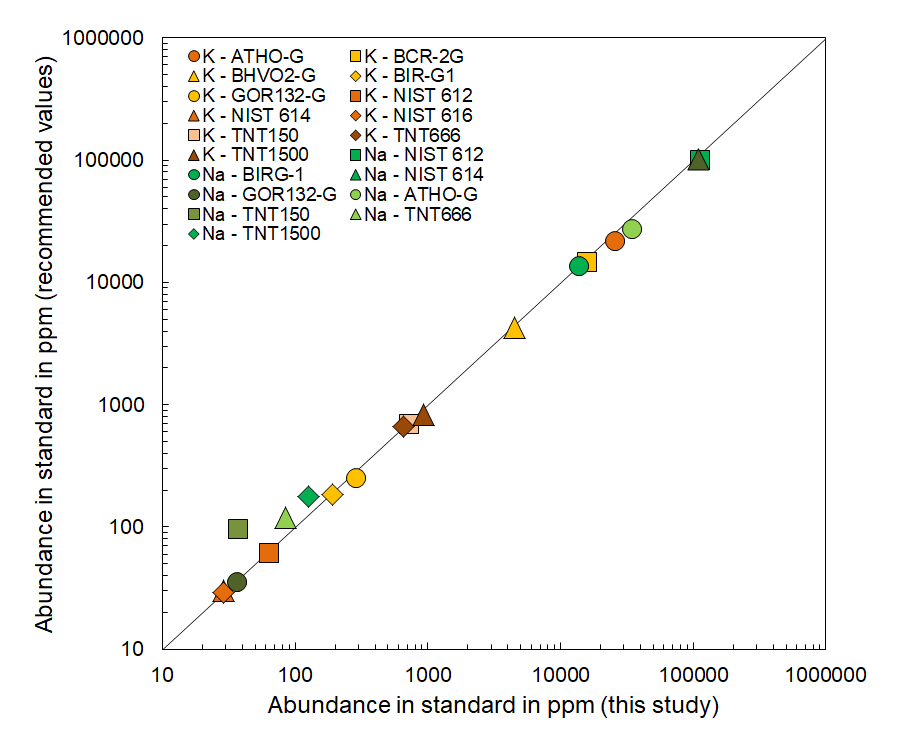


**Figure S.3.** Comparison of K and Na abundances in silicate reference materials derived with LA-ICP-MS versus the preferred K and Na abundances from the GeoRem website (accessible via <georem.mpch-mainz.gwdg.de/>*).* The 1SD errors are smaller than symbol sizes in all cases and are based on at least >5 analyses per reference material. The offset of the TNT reference materials is likely the result from uncertainties on previous analyses (e.g., EPMA detection limits) and is discussed in more detail in the section Quantification of K and Na abundances using LA-ICP-MS.


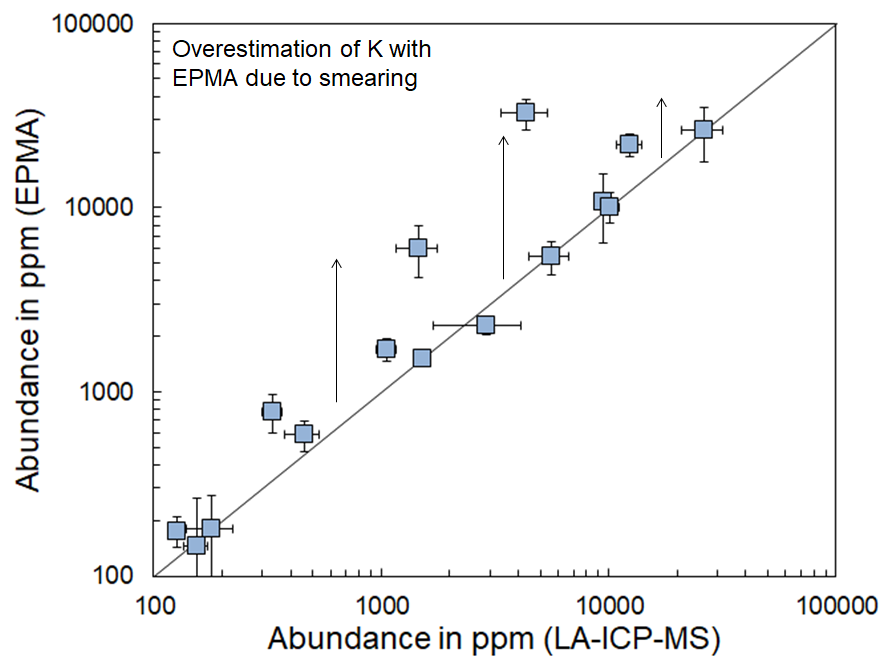


**Figure S.4.** Comparison of K abundances derived with EPMA and LA-ICP-MS. The consistent overestimation of K abundances from EPMA (by up to several wt.%, indicated by vertical arrows) relative to LA-ICP-MS values suggest K was preferentially smeared on to the sulfides due to dry polishing (see also Fig. 1 in main text file).


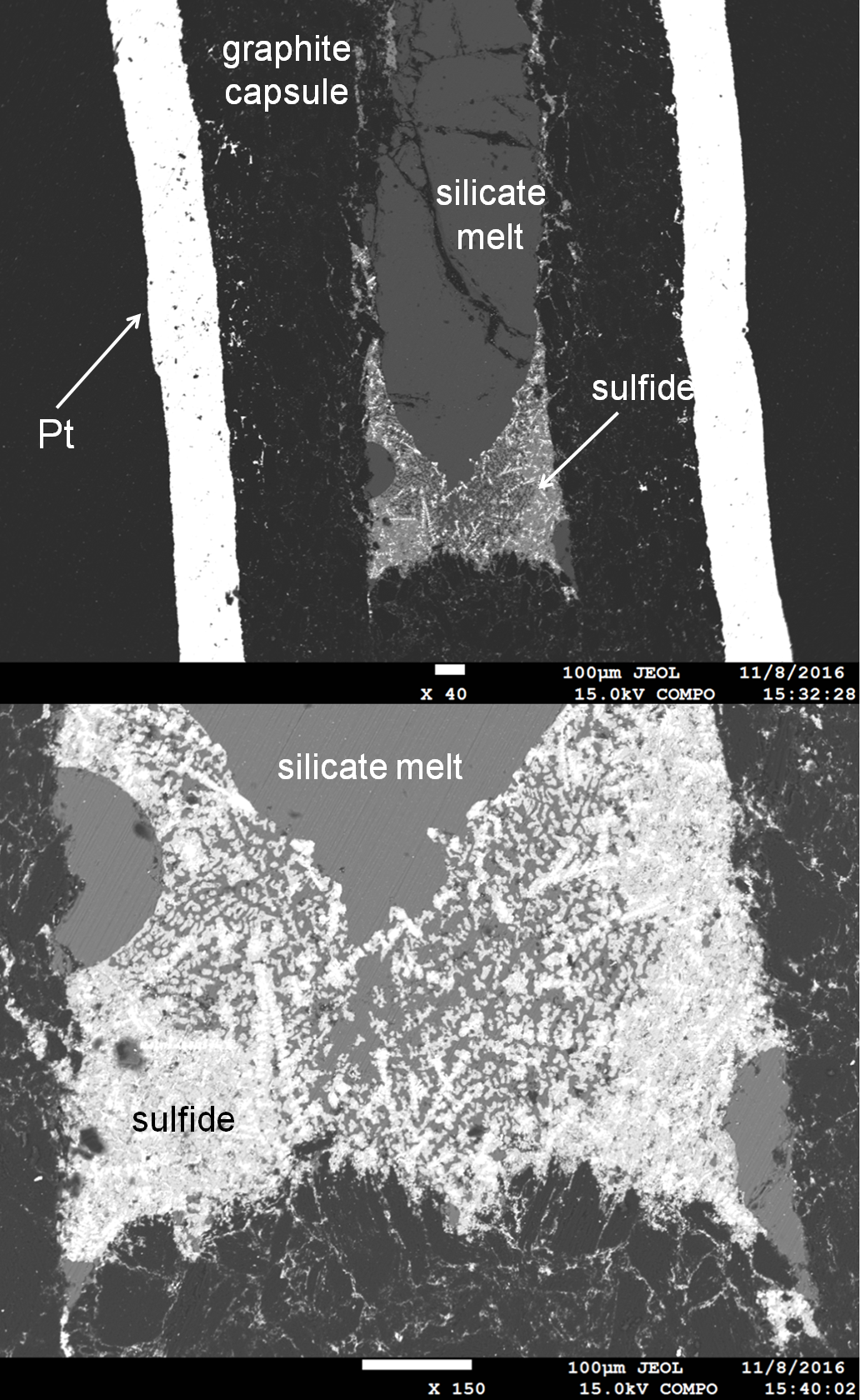


**Figure S.5**. Backscattered electron image of run product GGK6, where the sulfide (white) contained some silicate melt inclusions (grey) (see main text).


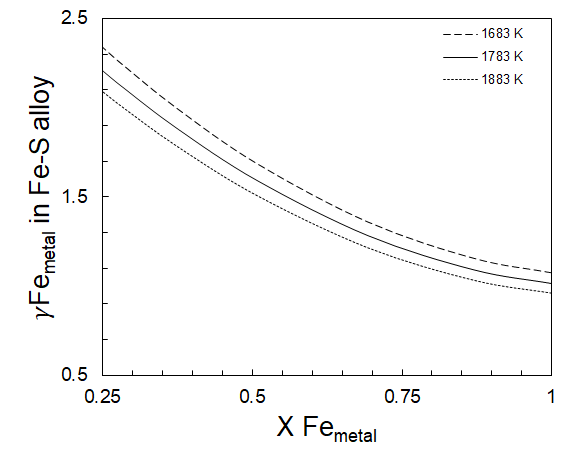


**Figure S.6**. The modeled effect of S on the activity coefficient of Fe in the Fe-S alloy for different run temperatures using the model of Lee and Morita^20^.


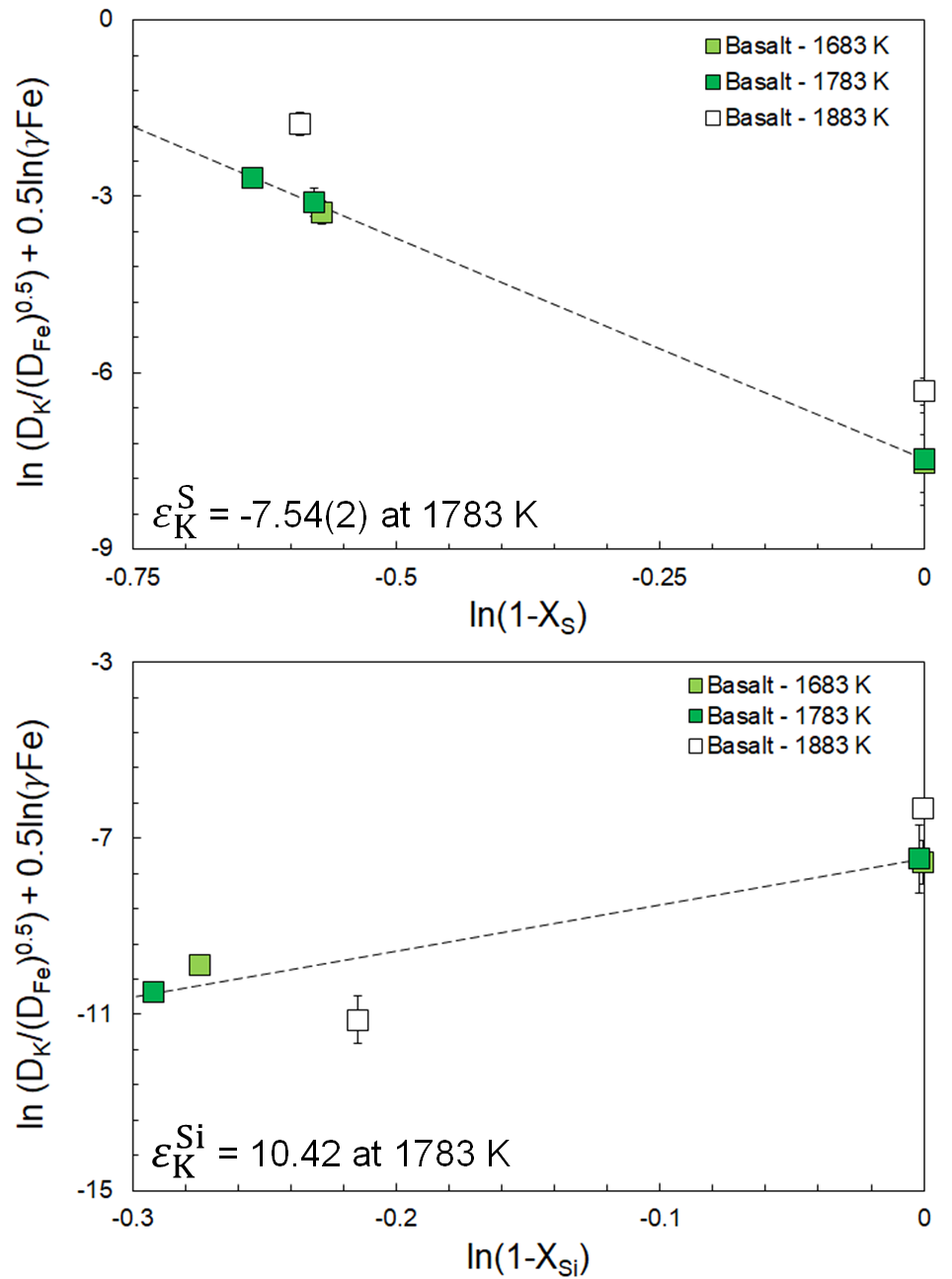


**Figure S.7**. The effect of dissolved sulfur and silicon in metal on exchange coefficients of K, where the epsilon value ($\varepsilon_{K}^{S, Si}$) represents the slope of the trend line. Errors are 2SE and were calculated using simple error propagation.

**
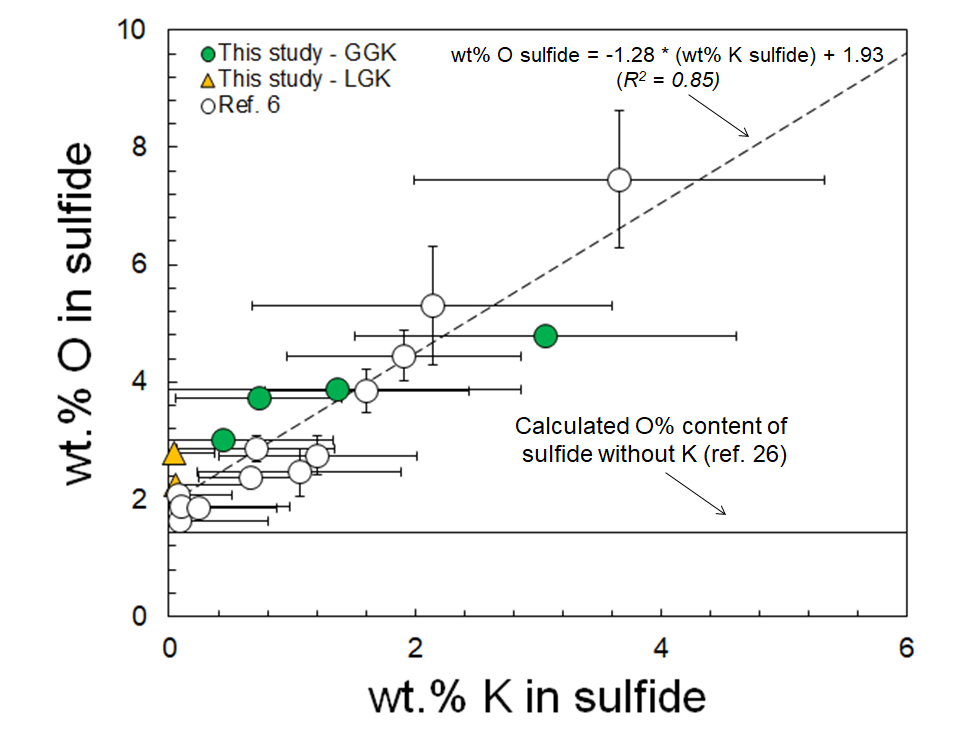
**

**Figure S.8**. Correlation between K and O (wt%) in metal (wt.% O sulfide = 1.28 * (wt.% K in sulfide) + 1.93). Errors are 1 standard deviation. Oxygen analyses are from this study (filled circles and triangles) and from the previous experiments of Murthy et al.^6^ (open circles). Horizontal line is the estimated O content of 1.44 wt.% for FeS at 1673 K for a silicate melt with 6 wt.% FeO calculated using the expression from Kiseeva and Wood^26^. The amount of O is greatly increased with increasing K.


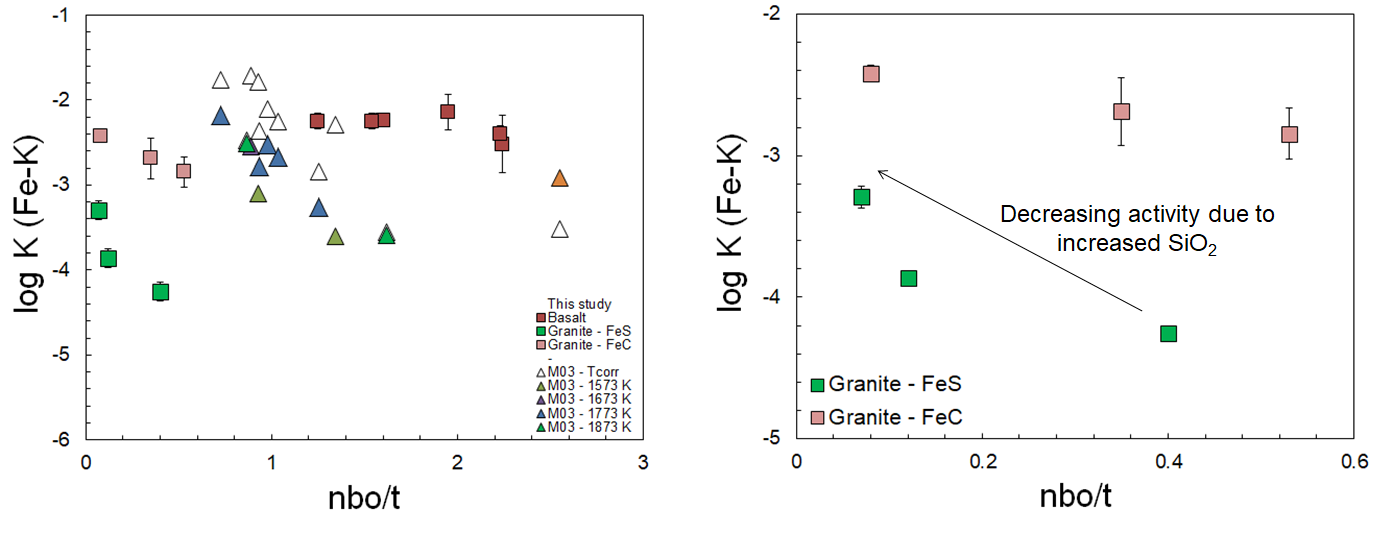


**Fig S.9.** The effect of silicate melt polymerization on $\log K_{K}$. Included is the data from Murthy et al.^6^, which was corrected using the interaction coefficients obtained in this study for K in Fe-S alloys (see main text).

**
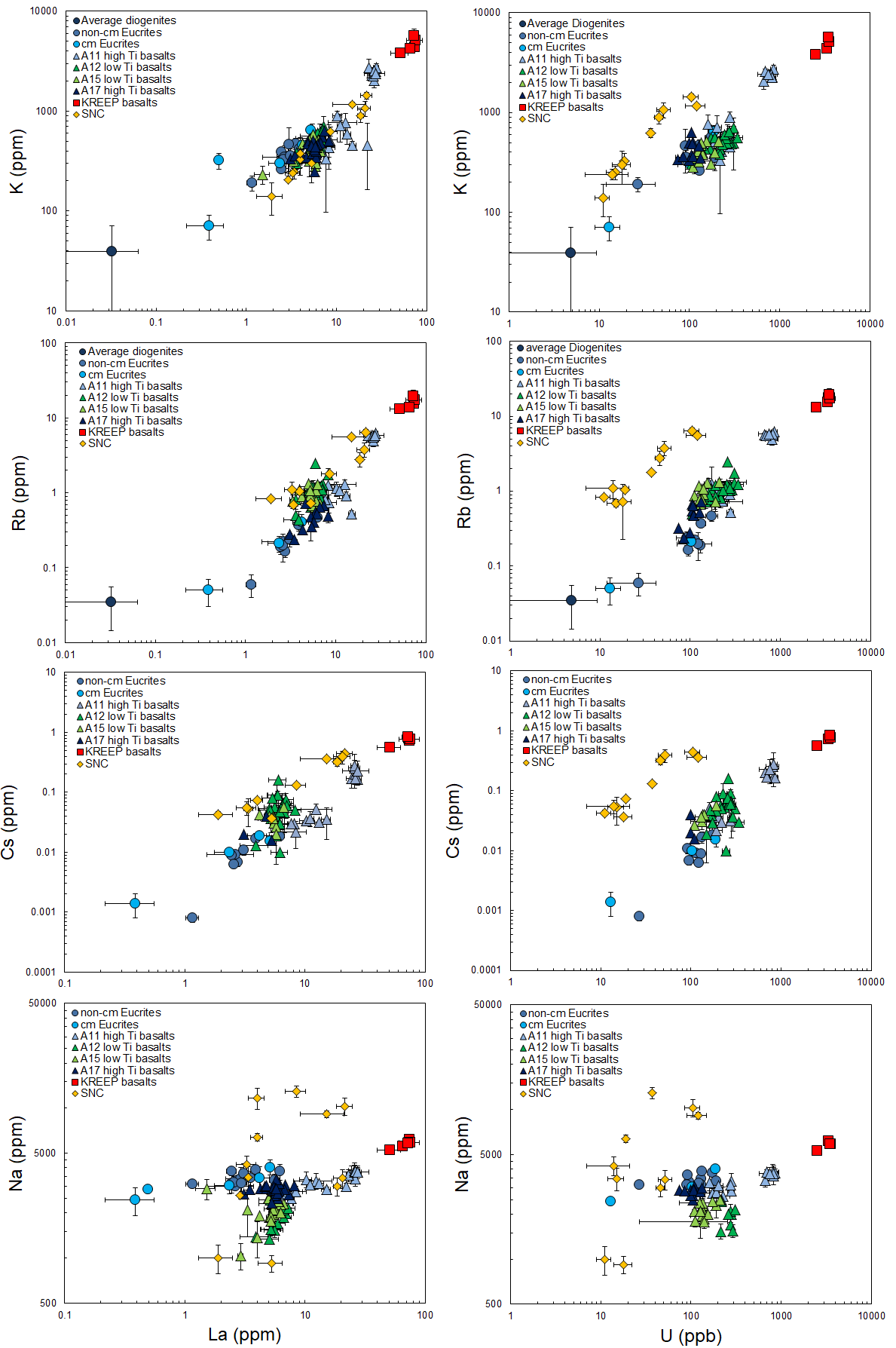
**

**Figure S.10.** Correlation between alkalis and incompatible refractory elements. Lunar data from the Lunar Sample Compendium^107^*,* eucrite data from Kitts and Lodders (1998)^37^, diogenite data from Jochum and Palme (1990)^108^, Birck and Allegre (1981)^91^, Kreutzberger et al. (1986)^90^, Takahashi and Masudat (1990)^109^, Mittlefehldt (1994)^110^, Mittlefehldt et al. (2012)^111^ and SNC data from Lodders (1998)^38^*.* Errors are 1 SD. Errors on diogenites are larger than usual due to incorporation of trace amounts of residual liquids in some diogenites.

**
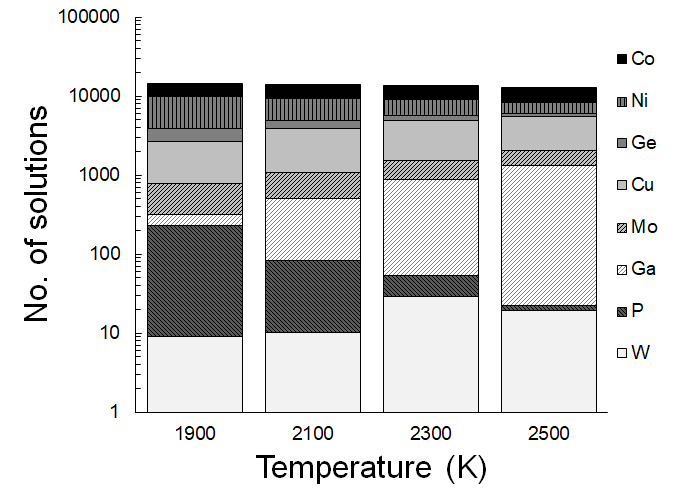
**

**Figure S.11.** The solution space for P, Co, Ni, Cu, Ga, Ge, Mo and W with variable temperature, assuming an oxidation state of -2.2±0.2 log units below the iron-wüstite buffer during core formation in Vesta and a 15 wt.% S bearing Vestan core for a H-chondrite + < 22% CV bulk composition.

**Table S.1.** Starting composition of silicate melts determined by EMPA and LA-ICP-MS

| **Run #** | **SiO_2_** | **Na_2_O** | **TiO_2_** | **Cr_2_O_3_** | **K_2_O** | **CaO** | **MgO** | **Al_2_O_3_** | **FeO** | **MnO** | **Total** | ***N*** |
| --- | --- | --- | --- | --- | --- | --- | --- | --- | --- | --- | --- | --- |
| **GGK** | 42.0(1) | 0.03(1) | 0.26(1) | 0.30(1) | 10.2(1) | 7.4(1) | 17.1(1) | 7.3(1) | 13.1(1) | 0.22(1) | 98.0(1) | 18 |
| **LGK** | 70.0(2) | 0.55(2) | 0.36(1) | - | 8.0(1) | 1.8(1) | 0.36(1) | 14.9(1) | 2.2(1) | 0.04(1) | 98.3(1) | 33 |

**Table S.2**. Calculated activity coefficients at the listed temperatures and corresponding oxygen fugacity values (relative to the iron-wüstite buffer or ΔIW). Effects of temperature on activity coefficients were incorporated using Eq. S.1.

| **Run** | T (K) | ΔIW | $\gamma_{\mathrm{FeO}}^{\mathrm{silicate}}$^19^ | $\gamma_{\mathrm{Fe}}^{\mathrm{metal}}$^18,20^ | $\gamma_{K}^{\mathrm{metal}}$ | $\gamma_{\mathrm{Na}}^{\mathrm{metal}}$ |
| --- | --- | --- | --- | --- | --- | --- |
| **GGK1** | 1683 | -1.46 | 0.64 | 0.73 | 1.99 | - |
| **GGK2** | 1783 | -1.13 | 0.70 | 0.73 | 1.97 | - |
| **GGK3** | 1883 | -1.29 | 0.67 | 0.72 | 1.96 | - |
| **GGK4** | 1683 | -2.63 | 0.70 | 1.84* | 0.05 | 0.05 |
| **GGK5b** | 1783 | -2.85 | 0.66 | 1.77* | 0.06 | 0.06 |
| **GGK6** | 1883 | -2.90 | 0.67 | 1.76* | 0.07 | 0.07 |
| **GGK7** | 1683 | -2.76 | 0.64 | 0.53 | 1.44 | - |
| **GGK8** | 1783 | -5.34 | 0.69 | 0.51 | 1.39 | - |
| **GGK9** | 1883 | -5.94 | 0.67 | 0.58 | 1.56 | - |
| **LGK1b** | 1883 | -2.55 | 1.02 | 1.56 | 0.09 | 0.09 |
| **LGK2** | 1683 | -3.06 | 1.01 | 1.75 | 0.05 | - |
| **LGK3b** | 1783 | -2.77 | 1.11 | 1.67 | 0.04 | - |
| **LGK4b** | 1683 | -2.83 | 1.06 | 0.72 | 1.97 | - |
| **LGK5** | 1783 | -1.24 | 0.98 | 0.73 | 1.98 | - |
| **LGK6** | 1883 | -0.94 | 0.98 | 0.72 | 1.96 | - |

*Calculated using model of Lee and Morita^20^

**Table S.3**. Composition of silicate melts determined by EPMA and LA-ICP-MS. Numbers in parentheses are 2 SE.

| **Run #** | **LGK-1b** | | **LGK-2** | | **LGK-3b** | | **LGK-4** | **LGK-4B** | **LGK-5** | | **LGK6** | **GGK-1** | **GGK-2** | **GGK-3** | **GGK-4** | **GGK-5b** |
| --- | --- | --- | --- | --- | --- | --- | --- | --- | --- | --- | --- | --- | --- | --- | --- | --- |
|  |  | |  | |  | |  |  |  | |  |  |  |  |  |  |
| **EPMA (wt%)** | *N = 13* | | *N = 13* | | *N = 5* | | *N = 11* | *N = 17* | *N = 29* | | *N = 17* | *N = 20* | *N = 16* | *N = 14* | *N = 17* | *N = 13* |
| **MgO** | 0.50(1) | | 0.47(1) | | 0.48(3) | | 0.37(1) | 0.52(1) | 0.47(1) | | 0.41(1) | 13.57(402) | 12.65(163) | 15.22(42) | 11.91(12) | 18.36(9) |
| **SiO_2_** | 68.68(47) | | 73.08(34) | | 70.69(36) | | 66.91(52) | 72.08(35) | 63.33(26) | | 59.20(51) | 40.16(70) | 37.07(20) | 38.56(8) | 48.65(26) | 47.20(17) |
| **Al_2_O_3_** | 12.03(15) | | 12.15(15) | | 12.44(11) | | 11.24(16) | 12.08(12) | 10.94(8) | | 9.97(20) | 6.77(98) | 6.64(41) | 6.27(12) | 9.30(4) | 7.60(4) |
| **CaO** | 1.36(1) | | 1.37(4) | | 1.25(3) | | 1.07(3) | 1.66(4) | 1.33(4) | | 1.16(1) | 6.80(99) | 6.51(63) | 6.42(18) | 8.91(8) | 7.57(4) |
| **FeO** | 4.55(14) | | 2.59(19) | | 3.09(23) | | 8.39(34) | 2.16(8) | 15.08(26) | | 21.10(15) | 19.09(42) | 25.41(44) | 22.23(9) | 6.49(4) | 5.07(11) |
| **K_2_O** | 6.51(4) | | 7.41(6) | | 6.80(7) | | 7.04(5) | 7.33(5) | 6.39(2) | | 5.98(4) | 10.25(165) | 8.44(69) | 8.17(14) | 9.16(3) | 7.82(10) |
| **Na_2_O** | 0.58(2) | | 0.65(1) | | 0.73(1) | | 0.57(1) | 0.69(2) | 0.67(2) | | 0.63(2) | 0.18(3) | 0.14(2) | 0.12(1) | 0.26(1) | 0.09(1) |
| **MnO** | 0.03(1) | | 0.03(1) | | 0.03(1) | | 0.02(1) | 0.07(1) | 0.03(1) | | 0.02(1) | 0.18(1) | 0.19(1) | 0.19(1) | 0.11(1) | 0.08(1) |
| **Cr_2_O_3_** | b.d.l. | | b.d.l. | | b.d.l. | | b.d.l. | 0.15(2) | b.d.l. | | b.d.l. | 0.18(1) | 0.29(2) | 0.14(1) | 0.08(1) | 0.04(1) |
| **TiO_2_** | 0.33(1) | | 0.36(1) | | 0.27(1) | | 0.31(1) | 0.35(1) | 0.33(1) | | 0.29(1) | 0.24(4) | 0.25(2) | 0.24(1) | 0.34(1) | 0.28(1) |
| **SO_2_** | 0.37 (14) | | 0.01(1) | | 0.63(18) | |  |  |  | |  |  |  |  | 0.63(2) | 0.98(10) |
| **Total** | 94.95(45) | | 98.11(31)^a^ | | 96.42(43) | | 95.96(57)^a^ | 97.12(38)^b^ | 98.58(23) | | 98.77(40) | 97.42(32) | 97.49(31) | 97.56(11) | 95.84 (21) | 95.15(23) |
| **Total ^c^** | 96.30(45) | | 99.09 (31) | | 97.64(43) | | 96.70(57) | 97.45(38) | - | | 99.36(40) | 99.10(32) | 99.33(31) | 99.30(11) | 98.48(21) | 96.56(23) |
| **LA-ICP-MS** | *N = 2* | | *N = 3* | | *N = 4* | | *N = 2* | *N = 2* | *-* | | *N = 2* | *N = 3* | *N = 4* | *N = 4* | *N = 4* | *N = 8* |
| **CaO (wt.%)** | 1.44(2) | | 1.33(5) | | 1.05(2) | | 1.00(3) | 1.40(28) | - | | 1.11(15) | 5.51(27) | 6.45(30) | 6.67(28) | 9.01(90) | 7.43(16) |
| **K_2_O** | 7.76(21) | | 8.38(3) | | 8.06(10) | | 7.92(14) | 7.83(33) | - | | 6.59(79) | 9.63(77) | 10.27(70) | 9.91(20) | 11.80(101) | 9.29(20) |
| **Na (ppm)** | n.d.^d^ | | n.d. | | 5911(4) | | n.d. | n.d. | - | | 4659(654) | 974(66) | 1034(84) | 869(35) | 1593(258) | 676(36) |
| **Ti** | 1875(148) | | 2102(23) | | 1399(36) | | 1799(13) | 1942(66) | - | | 1735(19) | 1149(58) | 1385(99) | 1382(31) | 1956(236) | 1521(46) |
| **Cr** | 23(1) | | 43(2) | | 16(2) | | 23(2) | 1838(725) | - | | 29(4) | 1217(96) | 1137(58) | 839(22) | 700(86) | 9.09(140) |
| **Mn** | 245(5) | | 239(1) | | 166(9) | | 172(5) | 542(65) | - | | 200(13) | 1431(155) | 1355(48) | 1375(21) | 1015(78) | 623(34) |
| **Run #** | **GGK-6** | | **GGK-7** | | **GGK-8** | | **GGK-9** |  |  | |  |  |  |  |  |  |
| **EPMA (wt%)** | *N = 30* | | *N = 6* | | *N = 20* | | *N = 27* |  |  | |  |  |  |  |  |  |
| **MgO** | 18.47(6) | | 8.86(218) | | 15.27(43) | | 16.87(35) |  |  | |  |  |  |  |  |  |
| **SiO_2_** | 48.97(8) | | 62.85(206) | | 56.55(23) | | 58.13(63) |  |  | |  |  |  |  |  |  |
| **Al_2_O_3_** | 8.00(2) | | 8.84(125) | | 7.69(5) | | 7.05(9) |  |  | |  |  |  |  |  |  |
| **CaO** | 7.63(2) | | 4.49(172) | | 7.93(8) | | 7.43(13) |  |  | |  |  |  |  |  |  |
| **FeO** | 4.36(4) | | 2.74(62) | | 0.13(3) | | 0.08(1) |  |  | |  |  |  |  |  |  |
| **K_2_O** | 8.16(4) | | 11.14(142) | | 9.90(17) | | 8.38(7) |  |  | |  |  |  |  |  |  |
| **Na_2_O** | 0.12(1) | | 0.34(12) | | 0.51(5) | | 0.30(1) |  |  | |  |  |  |  |  |  |
| **MnO** | 0.08(1) | | 0.07(2) | | 0.05(1) | | 0.13(1) |  |  | |  |  |  |  |  |  |
| **Cr_2_O_3_** | 0.03(1) | | 0.04(3) | | b.d.l. | | b.d.l. |  |  | |  |  |  |  |  |  |
| **TiO_2_** | 0.29(1) | | 0.07(3) | | 0.05(1) | | 0.13(1) |  |  | |  |  |  |  |  |  |
| **SO_2_** | 1.19(3) | |  | |  | |  |  |  | |  |  |  |  |  |  |
| **Total** | 97.29(10) | | 99.46(45) | | 98.07(17) | | 98.51(10) |  |  | |  |  |  |  |  |  |
| **Total ^c^** | 99.89(10) | | 100.09(45) | | 98.95(17) | | 99.70(10) |  |  | |  |  |  |  |  |  |
| **LA-ICP-MS** | *N = 6* | | *N = 1* | | *N = 7* | | *N = 4* |  |  | |  |  |  |  |  |  |
| **CaO (wt.%)** | 8.88(61) | | 4.87(4) | | 7.22(30) | | 7.10(70) |  |  | |  |  |  |  |  |  |
| **K_2_O** | 10.76(77) | | 11.78(116) | | 10.78(87) | | 9.57(83) |  |  | |  |  |  |  |  |  |
| **Na (ppm)** | 910(6) | | 1768(200) | | 2882(884) | | 2053(104) |  |  | |  |  |  |  |  |  |
| **Ti** | 1868(122) | | 539(67) | | 467(231) | | 697(197) |  |  | |  |  |  |  |  |  |
| **Cr** | 251(19) | | 201(26) | | 24(12) | | 26(18) |  |  | |  |  |  |  |  |  |
| **Mn** | | 682(45) | | 525(66) | | 625(229) | 948(183) |  | |  |  |  |  |  |  |  |

**^a^** Totals do not include trace elements Ni, As, Se, Cd, In, Sb, Te, Pb ^b^ Totals do not include trace elements P, V, Ni, Cu, Ge, Mo, Sn, W ^c^ Totals based on K abundances from LA-ICP-MS ^d^ Not determined.

**Table S.4**. Composition of metallic melts determined by EPMA and LA-ICP-MS. Numbers in parentheses are 2 SE.

| **Run #** | **LGK-1b** | | **LGK-2** | | **LGK-3b** | | **LGK-4** | | **LGK-4B** | **LGK-5** | **LGK-6** | | **GGK-1** | **GGK-2** | **GGK-3** | **GGK-4** | **GGK-5b** |
| --- | --- | --- | --- | --- | --- | --- | --- | --- | --- | --- | --- | --- | --- | --- | --- | --- | --- |
| **EPMA** | *N = 20* | | *N = 16* | | *N = 14* | | *N = 6* | | *N = 44* | *N = 10* | *N = 20* | | *N = 9* | *N = 13* | *N = 17* | *N = 20* | *N = 8* |
| **Fe (wt.%)** | 62.12(31) | | 56.09(46) | | 59.63(45) | | 86.82(61) | | 81.75(44 | 91.94(53) | 93.27(36) | | 92.99(18) | 92.47(25) | 92.30(29) | 58.43(41) | 55.89(84) |
| **O** | 2.87(32) | | 1.99(2)^a^ | | 2.25(59) | | -. | | - | - | - | | - | - | - | 3.72(67) | 3.88(150) |
| **Si** | 0.04(1) | | 0.08(2) | | 0.02(1) | | 0.11(3) | | 0.76(4) | 0.02(1) | 0.02(1) | | 0.01(1) | 0.02(1) | 0.01(1) | 0.03(1) | 0.01(1) |
| **S** | 32.94(39) | | 29.44(26) | | 34.31(30) | | - | | - | - | - | | - | - | - | 33.31(38) | 33.90(61) |
| **Ni** | 0.03(1) | | 3.45(26) | | 0.02(1) | | 4.67(21) | | 1.77(9) | 0.01(1) | 0.01(1) | | b.d.l. | 0.02(1) | 0.01(1) | 0.02(1) | 0.02(1) |
| **Cu** | - | | - | | - | | - | | 0.30(3) | - | - | | - | - | - | - | - |
| **Cd** | - | | 0.19(10) | | - | | - | | - | - | - | | - | - | - | - | - |
| **In** | - | | 1.13(15) | | - | | - | | - | - | - | | - | - | - | - | - |
| **Sn** | - | | - | | - | | - | | 1.55(17) | - | - | | - | - | - | - | - |
| **Pt** | 0.88(44) | | 0.10(5) | | 1.39(53) | | 0.34(20) | | b.d.l. ^d^ | n.d.^e^ | n.d. | | n.d. | 0.07(4) | 0.17(6) | 0.32(20) | 0.35(15) |
| **Ca (ppm)** | 176(44) | | 7(12) | | 133(66) | | b.d.l. | | b.d.l. | n.d. | 33(23) | | n.d. | n.d. | 6(8) | 956(189) | 1364(284) |
| **Mg** | 94(22) | | 14(14) | | 199(62) | | b.d.l. | | b.d.l. | n.d. | 37(24) | | n.d. | n.d. | 101(99) | 1249(381) | 227(58) |
| **Ti** | 123(37) | | 63(32) | | 67(29) | | 92(57) | | b.d.l. | n.d. | 33(21) | | n.d. | n.d. | 67(38) | 20(15) | 71(34) |
| **Al** | 93(34) | | b.d.l.^b^ | | 81(32) | | b.d.l. | | 88(34) | n.d. | 47(25) | | n.d. | n.d. | 73(29) | 103(35) | 68(32) |
| **Cr** | 149(37) | | 312(66) | | 184(51) | | 16(21) | | 11368(524) | n.d. | n.d. | | n.d. | n.d. | 170(37) | 3413(1166) | 1451(208) |
| **Mn** | 522(67) | | 492(67) | | 472(62) | | 31(45) | | 132(32) | n.d. | n.d. | | n.d. | n.d. | 10(15) | 1418(105) | 1161(68) |
| **Na** | 133(43) | | 83(43) | | 370(85) | | 422(84) | | b.d.l. | n.d. | 122(94) | | n.d. | 71(58) | - | 166(62) | 195(44) |
| **K** | 176(37) | | 584(114) | | 783(186) | | 231(40) | | b.d.l. | b.d.l. | 7(8) | | b.d.l. | 35(25) | 180(92) | 5428(1106) | 21948(3166) |
| **Total** | 98.98(32) | | 93.70(42 )^b^ | | 95.55(34) | | 92.02(68)^b^ | | 87.33(32)^c^ | 91.97(53) | 93.30(35) | | 93.00(18) | 92.59(26) | 92.45(30) | 96.91(41) | 95.99 |
| **C ^g^** | - | | - | | - | | - | | - | 8.03(53) | 6.70(35) | | 7.00(18) | 7.41(26) | 7.55(30) | - | - |
| **X_C_ ^h^** | 0.05 | | 0.04 | | 0.02 | | 0.19 | | 0.19 | 0.20 | 0.21 | | 0.19 | 0.20 | 0.21 | 0.03 | 0.03 |
| **LA-ICP-MS** | *N = 17* | | *N = 3* | | *N = 11* | | *-* | | *N = 6* | *N = 12* | *N = 12* | | *N = 3* | *N = 10* | *N = 5* | *N = 3* | *N = 12* |
| **Fe (wt.%)** | 62.73(151) | | n.d. | | 56.90(150) | | - | | 72.74(420) | int. | int. | | int. | int. | int. | 55.77(825) | 58.67(306) |
| **Na (ppm)** | 39(5) | | n.d. | | 84(17) | | *-* | | n.d. | <35 | <26 | | <29 | <35 | 48(17) | 76(15) | 135(46) |
| **Si** | 557(69) | | 2791(2226) | | 329(46) | | *-* | | 8770(613) | 845(164) | 1189(335) | | b.d.l. | 630(114) | 912(298) | 541(145) | 675(192) |
| **K** | 127(9) | | 457(158) | | 332(34) | | - | | 53(14) | 26(11) | 39(14) | | 57(26) | 56(31) | 180(42) | 5584(1120) | 12380(1561) |
| **Ca** | 125(15) | | b.d.l. | | 165(22) | | - | | b.d.l. | b.d.l. | b.d.l. | | b.d.l. | b.d.l. | b.d.l. | 1139(137) | 1584(166) |
| **Ti** | 114(6) | | 148(19) | | 69(4) | | - | | 21(5) | 7.9(47) | 2.9(9) | | b.d.l. | b.d.l. | 4.1(12). | 23(1) | 22(3) |
| **Cr** | 191(6) | | 589(21) | | 179(4) | | - | | 8215(899) | b.d.l. | b.d.l. | | 113(6) | 210(50) | 174(31) | 2367(551) | 1366(73) |
| **Mn** | int. ^f^ | | 1050(42) | | int. | | - | | 90(4) | b.d.l. | b.d.l. | | 2.4(2) | 5.6(23) | 7.3(22) | int. | int. |
| **Ni** | 219(11) | | int. | | 206(10) | | - | | int. | 31(3) | 34(7) | | 31(5) | 37(11) | 34(4) | 160(39) | 205(14) |
| **Run #** | **GGK-6** | | **GGK-7** | | **GGK-8** | | **GGK-9** | |  |  |  | |  |  |  |  |  |
| **EPMA** | *N = 10* | | *N = 22* | | *N = 12* | | *N = 6* | |  |  |  | |  |  |  |  |  |
| **Fe (wt.%)** | 50.88(222) | | 80.67(24) | | 80.73(21) | | 84.35(27) | |  |  |  | |  |  |  |  |  |
| **O** | 4.79(155) | | - | | - | | - | |  |  |  | |  |  |  |  |  |
| **Si** | 0.01(1) | | 14.16(11) | | 15.31(12) | | 11.76(38) | |  |  |  | |  |  |  |  |  |
| **S** | 33.83(105) | | - | | - | | - | |  |  |  | |  |  |  |  |  |
| **Pt** | 5.87(173) | | 1.32(5) | | b.d.l. | | b.d.l. | |  |  |  | |  |  |  |  |  |
| **Ca (ppm)** | 564(185) | | 5(7) | | n.d. | | n.d. | |  |  |  | |  |  |  |  |  |
| **Mg** | 306(41) | | 176(148) | | n.d. | | n.d. | |  |  |  | |  |  |  |  |  |
| **Ti** | b.d.l. | | 322(72) | | n.d. | | n.d. | |  |  |  | |  |  |  |  |  |
| **Al** | 101(43) | | 49(24) | | n.d. | | n.d. | |  |  |  | |  |  |  |  |  |
| **Cr** | 1127(165) | | 820(57) | | n.d. | | n.d. | |  |  |  | |  |  |  |  |  |
| **Mn** | 1069(146) | | 3372(83) | | n.d. | | n.d. | |  |  |  | |  |  |  |  |  |
| **Na** | 293(116) | | 52(24) | | n.d. | | n.d. | |  |  |  | |  |  |  |  |  |
| **K** | 26311(8478) | | 25(10) | | b.d.l. | | b.d.l. | |  |  |  | |  |  |  |  |  |
| **Total** | 93.58(117) | | 95.91(51) | | 96.08(26) | | 96.15(25) | |  |  |  | |  |  |  |  |  |
| **C ^g^** | - | | 4.09(51) | | 3.92(26) | | 3.85(25) | |  |  |  | |  |  |  |  |  |
| **X_C_ ^h^** | 0.04 | | 0.07 | | 0.08 | | 0.11 | |  |  |  | |  |  |  |  |  |
| **LA-ICP-MS** | | *N = 2* | | *N = 10* | | *N = 14* | | *N = 9* |  |  | |  |  |  |  |  |  |
| **Fe (wt.%)** | | 41.21(166) | | 80.16(377) | | 90.27(1113) | | 76.79(984) |  |  | |  |  |  |  |  |  |
| **Na (ppm)** | | 188(37) | | <44 | | <53 | | <30 |  |  | |  |  |  |  |  |  |
| **Si** | | 21027(20268) | | int. | | int. | | int. |  |  | |  |  |  |  |  |  |
| **K** | | 20445(5443) | | 23(3) | | 52(6) | | 29(14) |  |  | |  |  |  |  |  |  |
| **Ca** | | 3562(6537) | | b.d.l. | | b.d.l. | | b.d.l. |  |  | |  |  |  |  |  |  |
| **Ti** | | 77(158) | | 239(20) | | 507(105) | | 465(52) |  |  | |  |  |  |  |  |  |
| **Cr** | | 1165(291) | | 775(36) | | 1211(305) | | 1274(282) |  |  | |  |  |  |  |  |  |
| **Mn** | | int. | | 3683(214) | | 4278(470) | | 3589(353) |  |  | |  |  |  |  |  |  |
| **Ni** | | 156(17) | | 576(48) | | 525(51) | | 449(47) |  |  | |  |  |  |  |  |  |

^a^ Calculated from correlation between measured O and K (Fig. S.8) **^b^** Totals do not include trace elements Ni, As, Se, Cd, In, Sb, Te, Pb ^c^ Totals do not include trace elements P, V, Ge, Mo, W ^d^ Below detection limit ^e^ Not determined ^f^ Used as internal standard ^g^ Based on EPMA totals ^h^ Carbon contents calculated using the online metal activity calculator^18^

**Table S.5**. Bulk planet and bulk mantle abundances of K and Na in the Moon, Mars and asteroid Vesta (all in ppm).

|  | **Core mass %** | **bulk Na** | **bulk mantle Na** | **required log D(Na)** | **bulk K** | **bulk mantle K** | **required log D(K)** |
| --- | --- | --- | --- | --- | --- | --- | --- |
| **Moon** | 1.5-2.5^100,101,112^ | 2591±130^113^* | 800^114^ |  | 165±135^115**^ | 36-38^116^ |  |
|  |  |  | 890^68^ |  |  | 56^68^ |  |
|  |  |  |  |  |  | 100^114^ |  |
| **Avg.** |  | 2591±130 | 845±45 | 2.03±18 | 165±135 | 68±32 | 1.97±0.85 |
| **Vesta** | 20-30^30,36,84,117^ | 6050±350^36^ | 3326±270^37*^ |  | 729±52^36^ | 596±179^93,94^ |  |
|  |  |  |  |  |  | 371±95^37**^ |  |
| **Avg.** |  | 5050±350 | 3326±270 | 0.64±17 | 729±52 | 512±236 | 0.29±0.72 |
| **Mars** | 20-25^31,34,42,117^ | 5770±574^33^ | 4000±800^32^ | 0.45±32 | 730±62^33^ | 309±36^32^ |  |
|  |  |  |  |  |  | 350±158*** |  |
| **Avg.** |  |  |  |  |  |  | 0.77±0.35 |

**Assuming BSE as bulk Moon* ***non-cumulate eucrites ***This study*
